# Characterization of HIV-1 integrase interaction with human Ku70 protein and initial implications for drug targeting

**DOI:** 10.1038/s41598-017-05659-5

**Published:** 2017-07-17

**Authors:** Andrey N. Anisenko, Ekaterina S. Knyazhanskaya, Artur O. Zalevsky, Julia Yu Agapkina, Aleksander I. Sizov, Timofey S. Zatsepin, Marina B. Gottikh

**Affiliations:** 1 Faculty of Bioengineering and Bioinformatics, Lomonosov Moscow State University, Moscow, Russia; 2 Chemistry Department, Lomonosov Moscow State University, Moscow, Russia; 3 Skolkovo Institute of Science and Technology, Skolkovo, Russia; 4 Belozersky Institute of Physico-Chemical Biology, Lomonosov Moscow State University, Moscow, Russia

## Abstract

Human Ku70/Ku80 protein is known to influence HIV-1 replication. One of the possible reasons may be the protection of integrase from proteasomal degradation by Ku70 subunit. We demonstrated that recombinant HIV-1 integrase and Ku70 form a stable complex, while no interaction of Ku70 with integrase from prototype foamy virus was observed. By analyzing protein subdomains we determined two binding sites in the structure of both Ku70 and integrase: the 51–160 a.a. region of integrase interacts with residues 251–438 of Ku70, whereas Ku70 N-terminal domain (1–250 a.a.) contacts an α6-helix in the 200–220 a.a. integrase region. Single substitutions within integrase (E212A or L213A) block the interaction with Ku70 thus indicating that the binding site formed by the 200–220 a.a. integrase region is crucial for complex formation. E212A/L213A substitutions decreased the integrase capacity to bind Ku70 in HEK293T cells. A conjugate of 2′-ОMe-GGUUUUUGUGU oligonucleotide with eosin is shown by molecular modeling to shield integrase residues E212/L213 and is effective in blocking complex formation of Ku70 with integrase what makes the complex between α6-helix and Ku70(1–250) a possible target for drug development.

## Introduction

Human immunodeficiency virus requires many cellular factors in order to successfully complete its replication^1^. Identification of the host cell factors that mediate these steps and determination of their role in HIV reproduction can lead to the discovery of new targets for HIV therapeutics that will overcome viral resistance to existing drugs^2, 3^. Among cellular proteins, Ku70 and/or Ku80 were identified as host partners for HIV-1^1, 4–9^. In human cell Ku70 and Ku80 form a heterodimeric complex named Ku antigen or Ku. As a component of DNA-dependent protein kinase (DNA-PK) the Ku heterodimer plays a key role in the non-homologous end joining (NHEJ) DNA repair by specifically binding DNA ends at the site of the lesion^10, 11^. In addition to NHEJ, Ku is involved in various cellular processes such as V(D)J recombination, AP-site repair, telomere maintenance, apoptosis, transcription and translation^12–17^.

Besides these important cellular functions, Ku is known to influence HIV-1 replication, although the exact mechanism remains obscure. In fact, there are several contradictory studies showing Ku participation in retroviral DNA integration^18–21^, in the transcription of integrated provirus^22–25^ and in functions of HIV-1 matrix protein^8^. It has also been found that DNA-PK triggers apoptosis in activated CD4+ T cells during early HIV infection^26^. Altogether, these data point to multiple roles of Ku in HIV-1 replication cycle and indicate the need for more detailed study of the effects of Ku that could confirm the significance of this protein as a novel probable target for antiretroviral therapy.

At least two possible explanations were proposed for the impact that Ku has on viral integration: participation as a member of DNA-PK complex in the repair of gaps resulting from the viral DNA integration into the cell genome^18, 27, 28^; and protection of HIV-1 integrase (IN) against proteasomal degradation^29^. IN catalyzes a covalent insertion of viral DNA produced by reverse transcription of the viral RNA into the chromosomes of infected cells; that is a crucial step in the retroviral life cycle^30, 31^. A variety of cellular proteins is considered as IN partners needed for the successful integration. Among them lens-epithelium-derived growth factor (LEDGF/p75) is the most studied partner of HIV-1 IN^32, 33^. The mode of IN/LEDGF binding is well characterized, inhibitors of this binding, named LEDGINs, are developed and their potential as anti-HIV drugs is shown^34, 35^. The latter fact indicates that the study of the HIV-1 IN interactions with its cellular partners is promising in the context of new antiretroviral drug development.

One of the Ku subunits, namely Ku70, is considered as a cellular partner for HIV-1 IN. Direct binding of Ku70 with IN was shown using the yeast two-hybrid screen and co-immunoprecipitation^5, 29^, but the biological relevance of the IN/Ku70 interplay is not entirely explicit. On one hand, it has been postulated that the IN/Ku70 binding protects IN against proteasomal degradation in human cells, and knockdown of Ku70 expression renders integration undetectable^29^. On the other hand, depletion of Ku80, which also decreased the intracellular level of Ku70, in transduced HCT 116 cells is not found to affect the efficiency of viral DNA integration into the cellular genome^23^.

Since Ku70 and Ku80 proteins participate in various cellular processes, the inconsistency of the results mentioned above might be explained by the influence of some cellular factors that are difficult to take into account. Therefore, we assumed that the best way to understand the biological significance of the IN/Ku70 interaction is through its inhibition that does not involve affecting Ku intracellular level or cellular functions. However, the inhibition approach requires the knowledge of the protein complex structure. Here using recombinant proteins we characterize the mode of HIV-1 IN binding with Ku70, and reveal regions within both proteins responsible for their interaction. We show that these proteins form a stable complex, which can be detected *in vitro*. The complex formation is secured by two sites, where one clearly is dominant deducing by the fact that the distortion of this site by amino acid substitutions or its blocking by the addition of a shielding molecule effectively abolishes complex formation between two proteins. Thus, our data provide the basis for rational design of inhibitors of the interaction between HIV-1 IN and Ku70.

## Results

### Recombinant Ku70 binds HIV-1 IN *in vitro*

**Figure 1:**
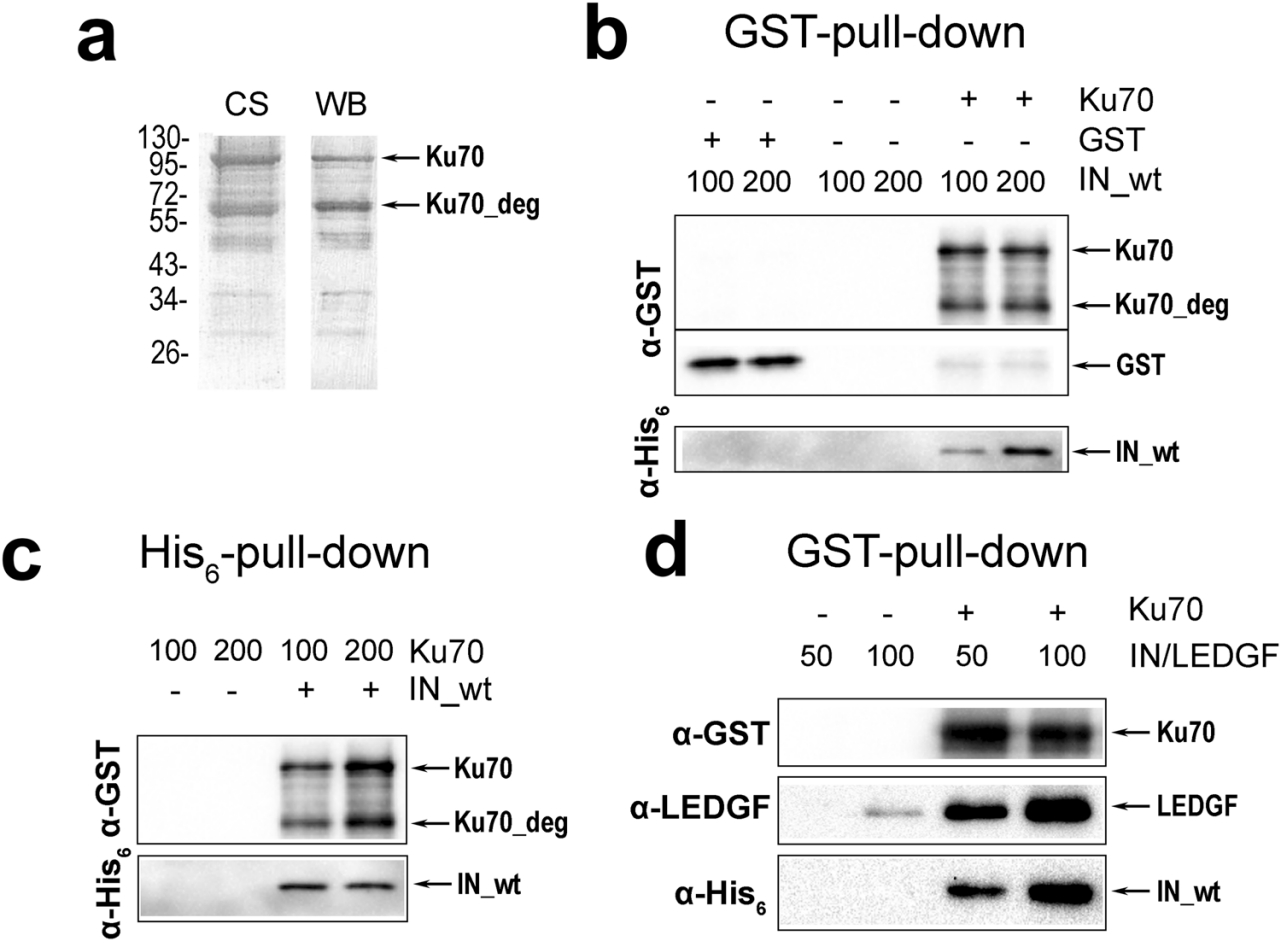
Analysis of the purity of recombinant preparation of Ku70 and its interaction with HIV-1 IN. (**a**) Recombinant N-GST-Ku70 protein after glutathione-sepharose purification analyzed by SDS PAGE with subsequent Coomassie blue staining (CS) and Western blot analysis with anti-GST antibody (WB). (**b**) Interaction between IN and Ku70 analyzed by a GST-pull-down assay. IN concentrations (nM) are marked above; Ku70 and GST were taken at 100 nM concentrations. (**c**) Interaction between IN and Ku70 analyzed by a His_6_-pull-down assay. Ku70 concentrations (nM) are marked above; IN was taken at 100 nM concentration. (**d**) Formation of the triple IN/LEDGF/Ku70 complex analyzed by GST-pull-down assay; 100 nM Ku70 was used, concentration of the preformed IN/LEDGF complex is marked above.

First we found out whether individual recombinant IN and Ku70 can form a stable complex *in vitro*. To this extent we expressed both proteins in *E.coli* and purified them by their N-terminal affinity tags that were His_6_ for HIV-1 IN and GST for Ku70 (Fig. S1). The later one showed a significant degree of degradation (Fig. 1a). Nevertheless, formation of a stable complex between recombinant Ku70 and IN was detected using a GST-pull down assay. GST domain alone did not interact with IN (Fig. 1b). An inverted experiment using a His_6_-pull down assay also showed a co-precipitation of Ku70 with IN while no interaction of Ku70 with Ni-NTA agarose was detected (Fig. 1c). Thus for the first time we showed a direct interaction of recombinant Ku70 and HIV-1 IN proteins that is consistent with Ku70 and IN forming a macromolecular complex^5, 29^.

The complex formation between IN and viral as well as cellular DNA is critical for integration. We tested the effect that a DNA mimicking the end of the viral U5 DNA has on the formation of a complex between IN and Ku70 and none was found (Fig. S2a). Moreover, formation of the triple complex DNA/IN/Ku70 was detected (Fig. S2b). Furthermore, we showed that IN in complex with LEDGF/p75 is still capable of complex formation with Ku70 (Fig. 1d). LEDGF/p75 transcription factor is an important positive IN-interacting component of the preintegration complex^33^.

### The role of the N-terminal part of Ku70 in complex formation with IN

**Figure 2:**
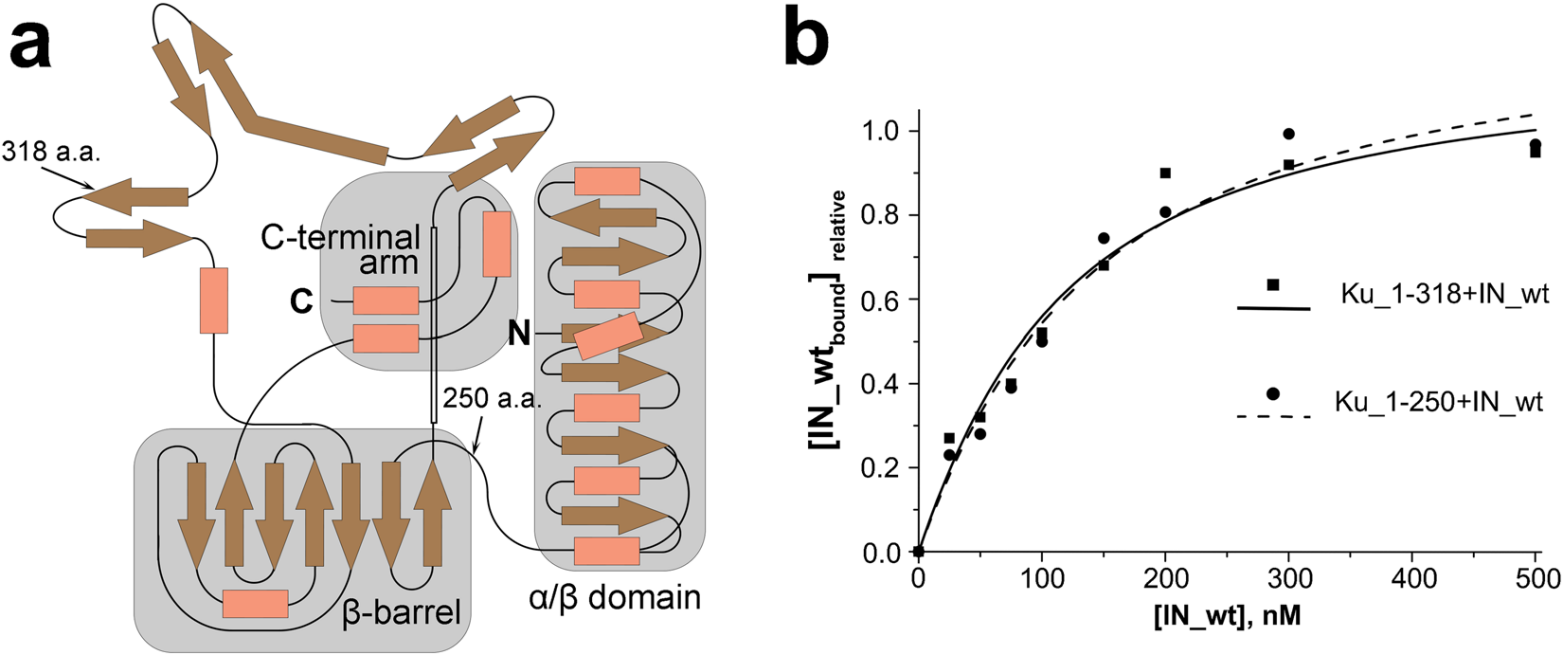
Design of deletion mutants of Ku70 and analysis of their IN-binding capacity. (**a**) Topology map of the secondary structure of Ku70 [35], C-termini of deletion mutants are marked by arrows. (**b**) GST-pull down assay analysis of the binding of Ku70_1-318 (■, solid line) and Ku70_1-250 (●, dashed line) with increasing concentrations of IN. Concentration of the Ku truncated mutants was 50 nM.

The full-length Ku70 consists of three independent domains: the N-terminal, the β-barrel with a DNA-binding loop and the helical C-terminal (Fig. 2a)^36^. In experiments with recombinant Ku70 we observed one major degradation product, which also co-precipitated with IN on Ni-NTA agarose as effectively as the full-length Ku70 (Fig. 1c). In gel it migrates at approx. 60 kDa, and the last identified C-terminal peptide resulting from the trypsin digestion was ^302^TFNTSTGGLLLPSDTKR^318^ as shown by mass-spectrometry (Table S1). According to these data, we suppose that the degradation product is the N-terminal fragment of Ku70 with an approximate length of 318 a.a. We individually expressed a deletion mutant Ku70_1-318 (Fig. 2a) containing an N-terminal GST-tag (Fig. S1a). An individual Ku70_1-318 also formed a stable complex with IN with a Kd = 90 ± 25 nM (Fig. 2b). The Ku70_1-318 deletion mutant contains the N-terminal domain and only two β-strands from the β-barrel structure (Fig. 2a). To elucidate a role of 250-318 a.a. forming two β-strands and the DNA-binding loop in formation and stabilization of the complex between Ku70 and IN, we constructed and purified a Ku70_1-250 mutant (Fig. S1a) that is the N-terminal α/β-domain of Ku70 (Fig. 2a). The GST-pull down assay showed that this mutant also formed a complex with IN as stable as its complex with Ku70_1-318 (Kd = 100 ± 30 nM). Hence, the N-terminal domain of Ku70 appears to be crucial for complex formation between these two proteins.

### Ku70 does not bind to the prototype foamy virus integrase

Previously it was shown that Ku70 interacts with an IN from Moloney murine leukemia virus (MoMLV)^5^. HIV and MoMLV belong to different genera of the *Orthoretrovirinae* subfamily, *i.e. Lentivirus* (HIV-1) and *Gammaretrovirus* (MoMLV). To check if the interaction between Ku70 and IN is characteristic for the whole subfamily, we tested Ku70 binding with an IN from a different retroviral subfamily of *Spumavirinae* - a prototype foamy virus (PFV). Of note, the most well-studied IN cellular co-factor LEDGF/p75 can only interact with lentiviral integrases but fails to form a complex with PFV or MoMLV INs^37, 38^.

**Figure 3:**
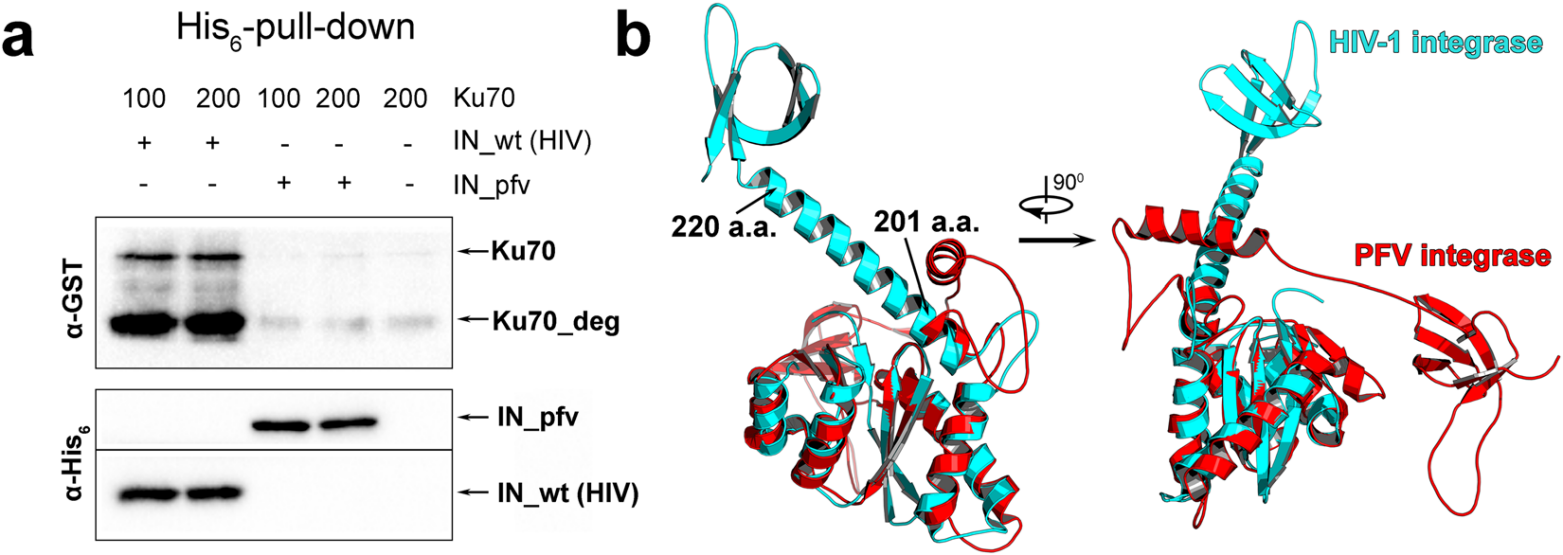
Ku70 interaction with IN HIV-1 and IN PFV. (**a**) His_6_-pull down assay for the binding of HIV-1 IN (IN_wt) or PFV IN (IN_pfv) with Ku70. Ku70 concentration (nM) marked above, both integrases taken at a 100 nM concentration. (**b**) Comparison of two-domain structures (catalytic + С-terminal domain) of HIV-1 IN (PDB ID: 1EX4) (turquoise) and PFV IN (PDB ID: 3OYI) (red).

We purified a recombinant N-terminally His_6_-tagged PFV IN (Fig. S1b) and analyzed its Ku70-binding activity in a His_6_-pull down assay. IN_pfv was unable to bind neither Ku70 nor its degraded variant Ku70_deg (Fig. 3a). A comparative analysis of the two-domain structure of the HIV-1 IN (catalytic + C-terminal domains, PDB ID: 1EX4) with its matching structural element from the IN_pfv (PDB ID: 3OYI) shows that major differences between the two structures start from residue 201 in the long helix α6 within HIV-1 IN (Fig. 3b). Accordingly, we suspected that the binding site for Ku70 is located in the helix α6 (a.a. 196–220).

### The catalytic domain of HIV-1 IN is involved in the interaction with Ku70

**Figure 4:**
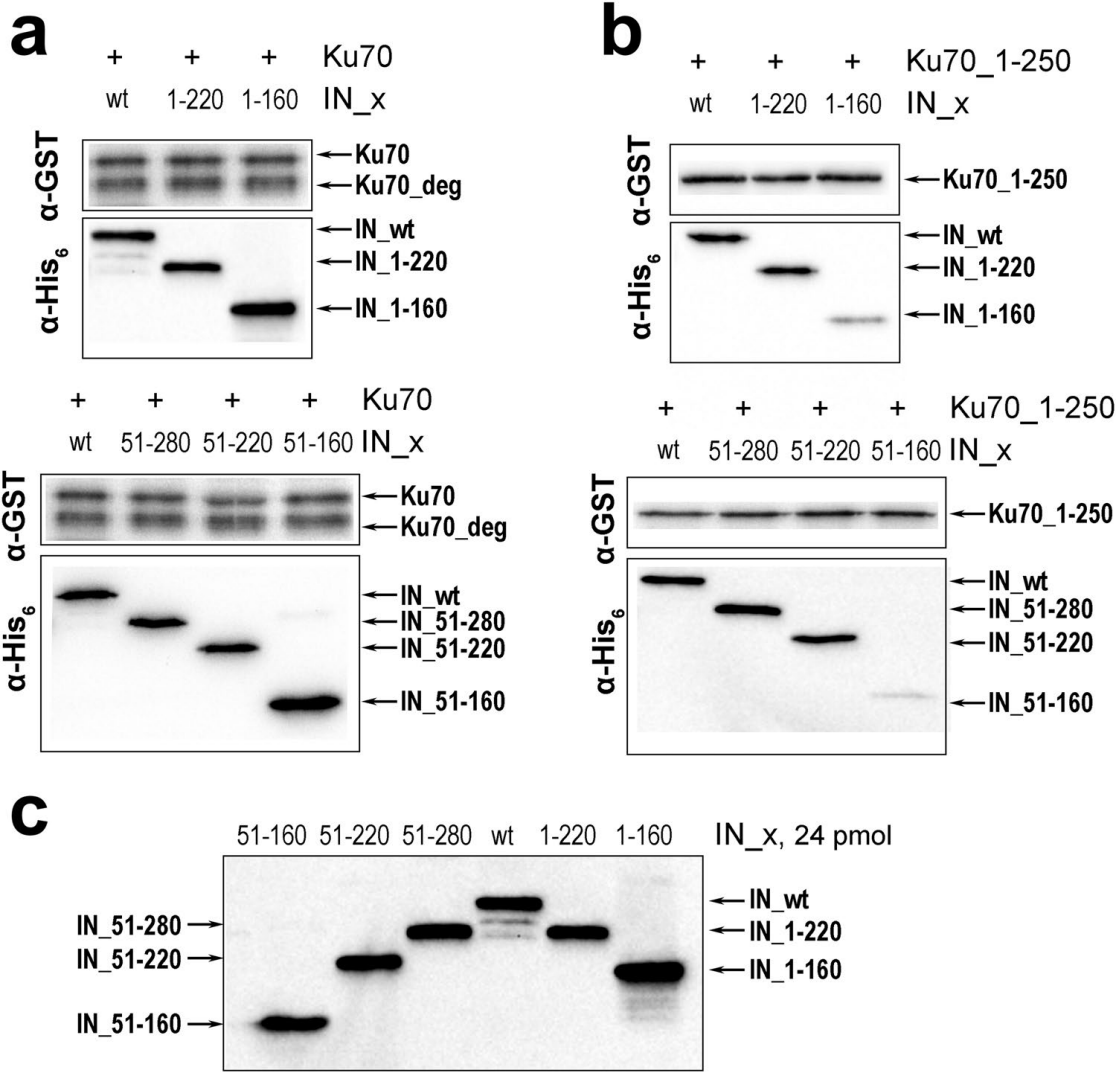
GST-pull down assay analysis of the binding of IN_wt and a series of its deletion mutants (200 nM) with full-length Ku70 (100 nM) containing a major degradation product (1–318 а.а.) (**a**) and with its truncated mutant Ku70_1-250 (100 nM) (**b**). Western blot analysis of the same amount (24 pmol) of different truncated IN mutants with anti-His_6_ antibody **(c)**.

Two IN mutants were constructed and expressed: IN_1-220 with truncated C-terminal domain and IN_1-160 that additionally lacked the helix α6 that we suspected to be involved in complex formation with Ku70 (Fig. S1b). In a GST-pull down assay both Ku70 and its mutant Ku70_1-250 formed a stable complex with IN_1-220 (Fig. 4a), which indicates that the C-terminal domain of IN is inessential for complex formation. Importantly, the Ku70_1-250 mutant can bind with equal efficiency both IN (Kd = 100 ± 30 nM, Figs 2b and 4b) and its mutant IN_1-220 (Kd = 120 ± 30 nM, Figs S2 and 4b) but not an IN_1-160 mutant (Fig. 4b). This result proves that the binding site for the N-terminal domain of Ku70 (a.a. 1–250) is located in the 160–220 a.a. region of IN that comprises the helix α6.

However, the 160–220 a.a. region is not the sole binding site, since Ku70 is capable of binding both the IN_1-220 and the IN_1-160 mutants (Fig. 4a). Therefore, a second binding site should exist that is responsible for the interaction between IN_1-160 and the region of Ku70 that locates within amino acids 251–609. To identify the second binding site we constructed and purified an individual N-terminal domain of IN (IN_1-50) as well as a series of truncation mutants that lack the N-terminal domain: IN_51-280, IN_51-220 and IN_51-160 (Fig. S1b). The IN_1-50 mutant failed to bind both Ku70 and its Ku70_1-250 mutant (Fig. S4). The lack of N-terminal domain did not affect the Ku70-binding activity of mutants IN_51-280, IN_51-220, IN_51-160 (Fig. 4). Interestingly, Ku70_1-250 formed complexes with IN_51-280 and IN_51-220 but failed to bind IN_51-160 (Fig. 4b). This result confirms the data by Zheng *et al*.^29^ that shows that the N-terminal domain is dispensable for complex formation between Ku70 and IN. We also showed that the second binding site is located in the IN region between residues 51 and 160.

### The core domain of Ku70 forms the second interaction site with IN

**Figure 5:**
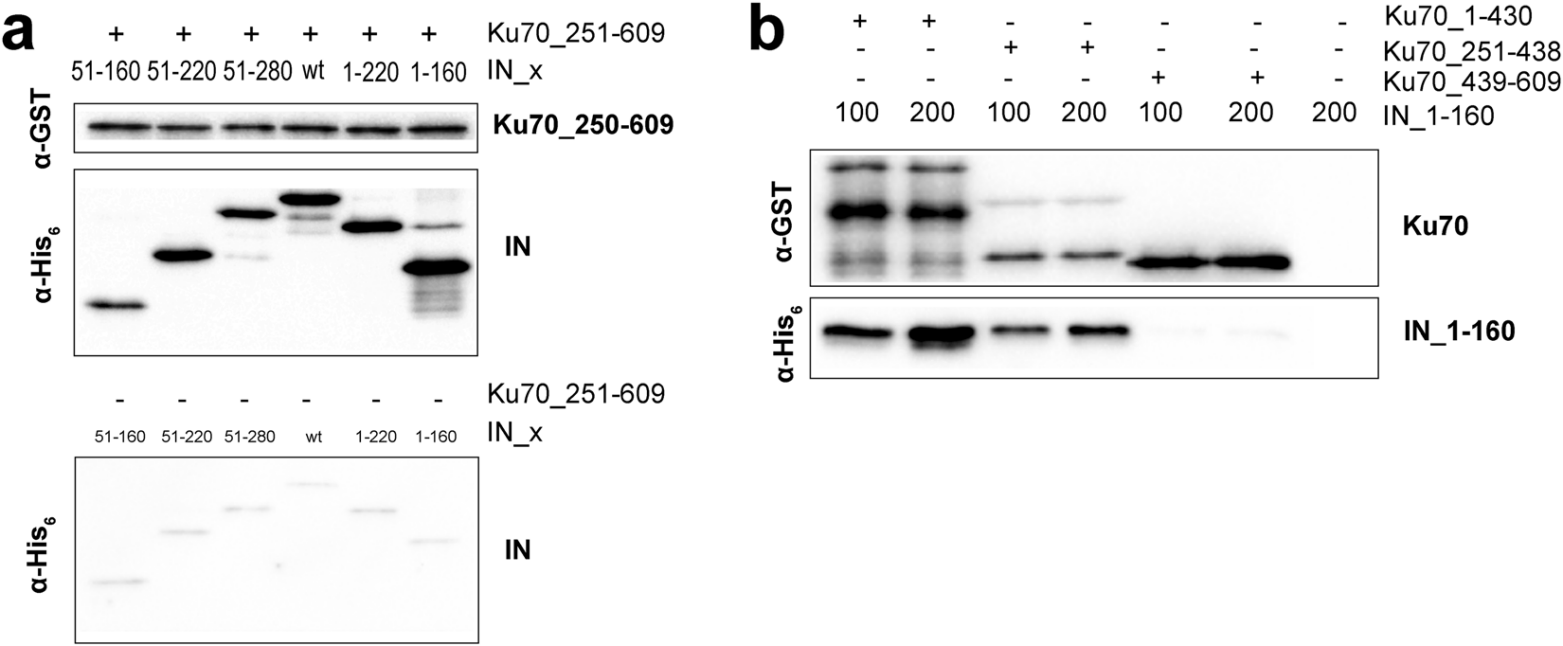
GST-pull down assay analysis of the binding between deletion mutants of Ku70 and IN. (**a**) Interaction between IN_wt and its deletion mutants (IN_1-220, IN_1-160, IN_51-280, IN_51-220, IN_51-160) (200 nM) with Ku70_250-609 (100 nM). The direct load of the truncated proteins shown at Fig. 4c. (**b**). Interaction between IN_1-160 (100, 200 nM) and Ku70_1-430, Ku70_251-438 or Ku70_439-609 (100 nM).

We constructed a truncated mutant Ku70_251-609 (Fig. S1a) in order to confirm the interaction of the C-terminal region of Ku70 with IN residues 51–160. This mutant formed stable complexes with all of IN mutants except the IN_1-50 protein (Figs 5a and S4), supporting the idea that Ku70 contains a second IN binding site that interacts with its 51–160 a.a. region.

Ku70_1-430 was shown to co-immunoprecipitate with HIV-1 IN upon overexpression of both proteins in HEK 293 T human cells^29^. In our hands, a recombinant mutant Ku70_1-430 (Fig. S1a) was capable of binding IN_1-160 (Fig. 5b). Together with detected interactions of Ku70_251-609 with both IN_1-160 and IN_51-160, this indicates that the second interaction site in Ku70 is located within a.a. 251-430. Ku70_251-438 and Ku70_439-609 mutants were constructed and expressed to prove this assumption (Fig. S1a). The Ku70_251-438 formed a stable complex with IN_1-160 whereas Ku70_439-609 failed to interact with it (Fig. 5b). This result clearly shows that IN region 50–160 interacts with the central domain of Ku70 (residues 251–438).

### Amino acid residues E212 and L213 of IN are crucial for complex formation with Ku70

**Figure 6:**
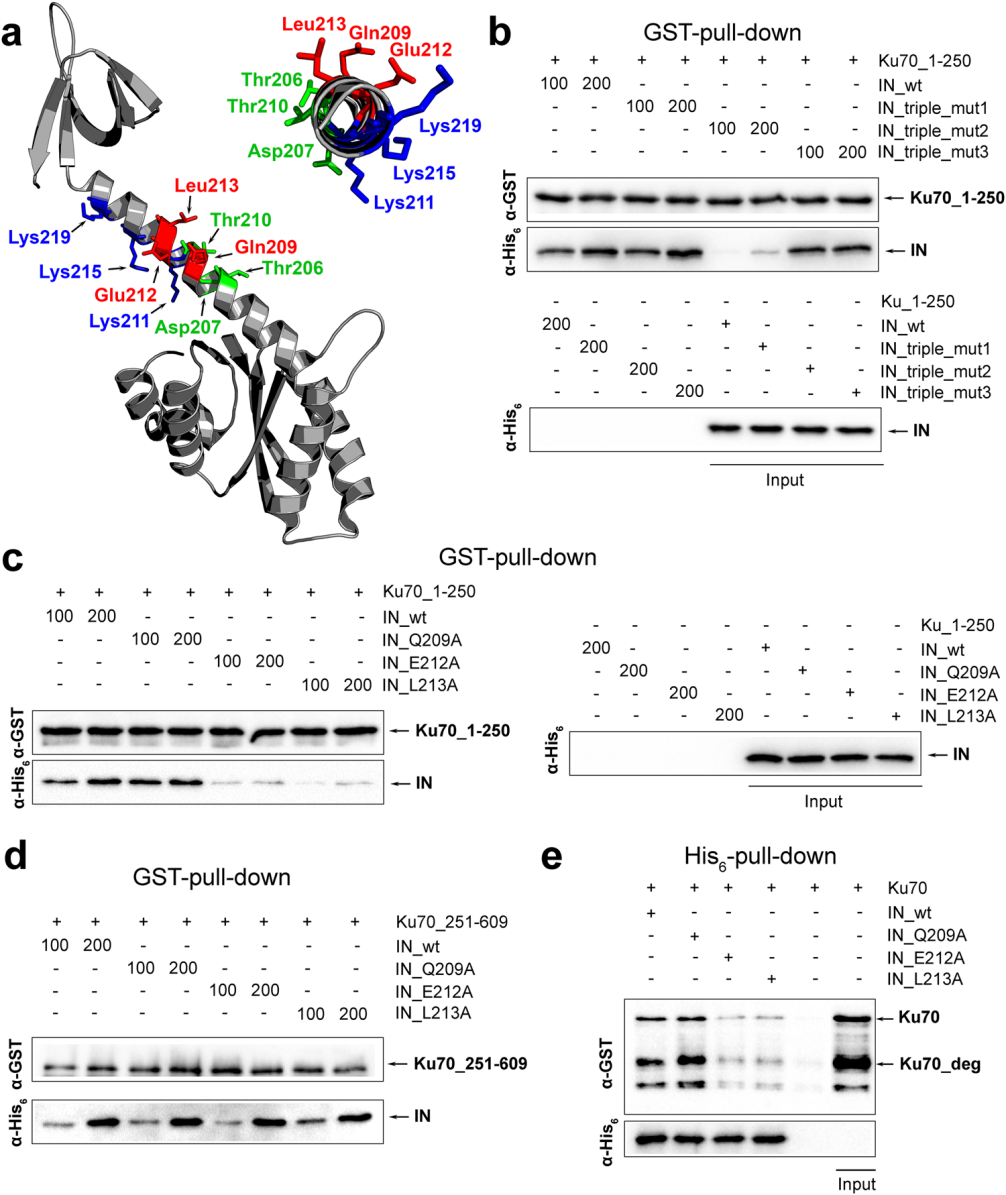
The influence of mutations in the 200–220 a.a. region of IN on its interaction with Ku70. (**a**) Structure of a two-domain region of IN (PDB ID: 1EX4); amino acid residues changed to alanine are marked by color. (**b**) GST-pull down assay analysis of the interaction between IN triple point mutants (100 and 200 nM) with Ku70_1-250 (100 nM). Cropped images are presented. Uncropped images are shown on Fig. S8. (**c**) GST-pull down assay analysis of the interaction between IN single point mutants (100 and 200 nM) with Ku70_1-250 (100 nM). (**d**) GST-pull down assay analysis of the interaction between IN single point mutants (100 and 200 nM) with Ku70_251-609 (100 nM). (**e**) His_6_-pull down assay analysis of the interaction between full-length Ku70 (200 nM) with IN single point mutants (100 nM).

We proceeded to study the binding center formed by IN residues 160–220. Considering that the helix α6 is important for complex formation, we constructed a set of IN triple point mutants: IN_Q209A/E212A/L213A, IN_T206A/D207A/T210A and IN_K211A/K215A/K219A. We started with three simultaneous alanine substitutions to increase the probability of interrupting IN/Ku70 interaction. The positions of substitutions were chosen so that the side chains of residues in each triad would face one side of the helix α6 (Fig. 6a). All triple point mutants were tested for their interaction with Ku70_1-250. Triple mutants IN_T206A/D207A/T210A and IN_K211A/K215A/K219A retained their Ku70-binding activity while the mutant IN_Q209A/E212A/L213A was unable to form a detectable complex with Ku70_1-250 (Fig. 6b). This result suggests that amino acids Q209, E212 and L213 could be forming the binding site between IN and Ku70_1-250.

We then constructed and expressed single point alanine mutants for each amino acid position in the triad Q209/E212/L213. The Q209A substitution did not alter the complex formation between IN and Ku70_1-250 while both IN_E212A and IN_L213A lost their affinity for Ku70_1-250 (Fig. 6c). Importantly, the same alanine substitutions did not affect the binding of IN with Ku70_251-609 that uses the second binding site (Fig. 6d).

To rule out that the loss of binding activity by the IN mutants could result from their misfolding, we tested the catalytic activity of three IN point mutants in 3′-processing and strand transfer reactions. The binding of the DNA substrate by IN occurs both in its catalytic and C-terminal domains^39, 40^. Since we introduced point mutations in the helix α6 that links these domains, any misfolding in the helix should cause a loss of the IN catalytic activity. All three point mutants retained their catalytic activity in both reactions although slightly decreased when compared to the wild type (Fig. S5). This proves that residues E212 and L213 are included in the first binding site and are crucial for the binding of IN with Ku70_1-250.

Importantly both IN_E212A and IN_L212A point mutants were also unable to form a complex with Ku70 as shown by a His_6_-pull down assay (Fig. 6e). This result proves that the first binding site is the dominant one in bimodular interaction model of HIV-1 IN and Ku70.

### A conjugate of an 11-mer oligonucleotide and eosin prevents complex forming between IN and Ku70

We suggested that the interactions between IN and Ku70 could be interrupted by blocking a region in IN between 200 and 220 a.a. Previously we developed an IN inhibitor that is a conjugate of 2′-O-methyl 11-mer GGUUUUUGUGU with eosin-Y (11-OM-E)^41, 42^. The inhibitory effect of 11-OM-E results from its capacity to destroy IN complex with the substrate DNA presumably interacting with the DNA-binding region within IN^43^. Here we demonstrated that 11-OM-E binds IN at its C-terminal domain (a.a. 220–270, Kd = 50 ± 10 nM as compared to the binding of 11-OM-E with full-length IN, Kd = 25 ± 5 nM). The catalytic domain of IN (a.a. 51–220) showed a very weak binding with 11-OM-E (Kd > 1 µM) while the N-terminal domain was incapable of 11-OM-E binding (Fig. S6). We assumed that if the α6-helix is indeed important for the IN interaction with Ku70 then by binding to the C-terminal domain the inhibitor could partially block the α6-helix by its oligonucleotide part and prevent the interaction.

**Figure 7:**
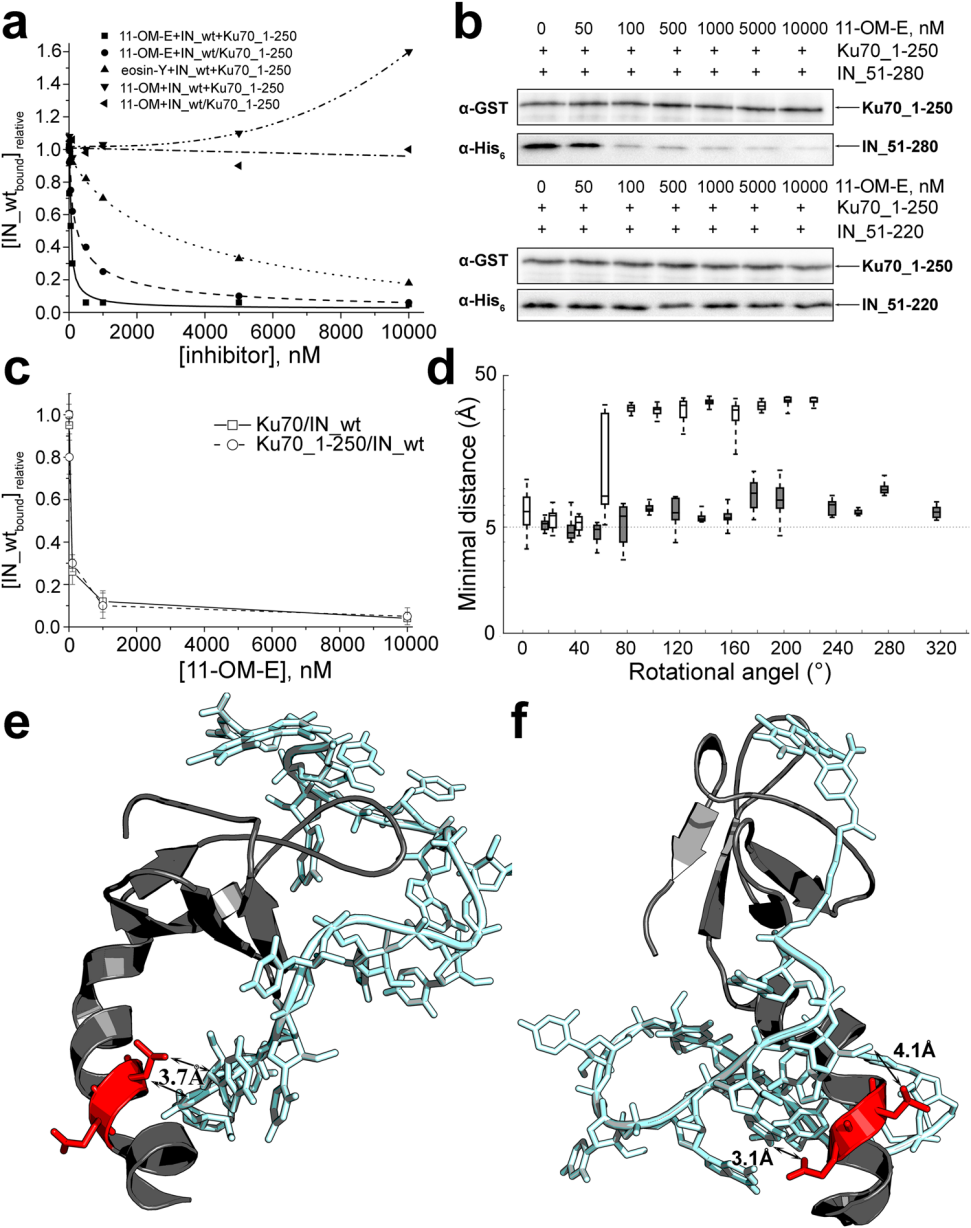
The interaction of an oligonucleotide inhibitor 11-OM-E with IN and its effect on the binding of IN with Ku70. (**a**) GST-pull down assay analysis of the influence that 11-OM-E and its fragments 11-OM and eosin-Y have on the formation of the complex between IN and Ku70_1-250 (curves “inhibitor+IN_wt+Ku70_1-250”) or on the stability of the preformed complex (depicted as “inhibitor+IN_wt/Ku70_1-250”). (**b**) GST-pull down assay analysis of the influence of increasing concentrations of 11-OM-E on the complex formation between IN deletion mutants IN_51-280 or IN_51-220 and Ku70_1-250. (**c**) GST-pull down assay analysis of the influence of increasing concentrations of 11-OM-E on the complex formation between IN and full-length Ku70 (□, solid line) or Ku70_1-250 deletion mutant (○, dashed line). (**d**) Distribution of minimal distances between atoms of the oligonucleotide part of 11-OM-E and the Е212 or L213 residues of IN. Results for the distal site are in white and for the proximal site in gray. (**e**,**f**) Possible arrangement modes for 11-OM-E when bound to IN. Eosin-Y is bound within distal site and the oligonucleotide reaches the L213 residue (3,7 Å) (**e**) or eosin is anchored in the proximal site and the oligonucleotide part reaches both E212 and L213 (2,8 Å и 4,1 Å) (**f**).

We started with analyzing the influence of 11-OM-E on the complex forming between IN and Ku70_1-250 deletion mutant that interacts with the helix α6 region. The efficiency of complex formation was significantly lowered in the presence of 11-OM-E (Fig. 7a), which shows that 11-OM-E inhibits the interaction between the N-terminal domain of Ku70 and IN with an IC_50_ = 50 ± 10 nM. The inhibition by 11-OM-E was significantly lowered when it was added to a preformed IN/Ku_1-250 complex (IC_50_ = 300 ± 40 nM, Fig. 7a). This indicates that 11-OM-E is a competitive inhibitor of Ku70_1-250 binding to IN.

To get a detailed understanding of the functional role of the oligonucleotide and eosin-Y parts of the conjugate, we separately tested the inhibiting activities of the 2′-O-methylated 11-mer GGUUUUUUGUGU (11-OM) and eosin-Y. The individual 11-OM did not affect the complex formation between IN and Ku70_1-250 but even had a stimulating effect at high concentrations. When added to a preformed complex, 11-OM did not alter the complex stability (Fig. 7a). Individual eosin-Y showed a weak inhibiting activity with an IC50 = 3 ± 1 µM (Fig. 7a). Thus only the conjugate of the oligonucleotide and eosin-Y can effectively block the complex formation between IN and Ku70.

To confirm that the inhibiting activity of 11-OM-E results from its binding to the C-terminal domain of IN accompanied by the helix α6 shielding, we tested its effect on complex formation of Ku70_1-250 with IN and its two deletion mutants: IN_51-280 and IN_51-220, where the later lacked the C-terminal domain. As expected, 11-OM-E showed a comparable inhibiting activity for Ku70_1-250 complex formation with IN and IN_51-280 but failed to inhibit the complex with IN_51-220, which did not contain the inhibitor binding site (Fig. 7b).

Thus, we showed that the 11-OM-E effectively inhibits the complex formation between IN and Ku70 by a competitive mechanism. It probably blocks the first binding site between IN region a.a. 200–220 and Ku70_1-250. Moreover, blocking of this site totally prevents complex formation between two full-length proteins (Fig. 7c). Altogether, our data prove that the first binding site is crucial for the interaction between two proteins, and any disturbance of this site impairs complex formation not only between IN and Ku70_1-250 but between two full-length proteins as well.

### Computer modelling of the interactions between 11-OM-E and IN

To further support our hypothesis that the helix α6 of IN is critical for complex formation between IN and Ku70, we performed the molecular modelling of the 11-OM-E interactions with IN. Taking into account that 11-OM alone does not inhibit the protein binding, we assumed that the correct position of inhibitor in the C-terminal part of IN is defined by eosin-Y that is conjugated to the oligonucleotide *via* linker (Fig. S7a). We performed a flexible docking of the eosin-Y + linker structure into the IN 206–270 a.a. fragment. This moiety can bind in two sites: a proximal site located closer to E212 and L213 and a distal site that is further removed (Fig. S7b). In each system the oligonucleotide part as 2′-OMe-RNA in A-form was added to eosin-Y+linker moiety followed by molecular dynamic simulations. The minimal distances from any atom in the oligonucleotide to any atom in E212 and L213 were estimated for each system (Fig. 7d) creating a number of predicted structures where the distance from the oligonucleotide to these residues falls into the 3.5–5.5 Å slot. This shows that despite the eosin-Y position the oligonucleotide part could reach the residues that are important for complex formation between IN and Ku70. During modeling we observed that the initial structure of the helix α6 was bent as a result of interactions with the oligonucleotide part of the inhibitor. The bending effect was most perceptible when eosin-Y was located in the distal site (Fig. S7b). Thus along with direct shielding of the the helix α6, 11-OM-E can induce structural changes in this region that lead to the interruption of IN/Ku70 complex formation.

### Substitution of amino acids E212 and L213 of IN impedes its interaction with Ku70 in 293 T cells

**Figure 8:**
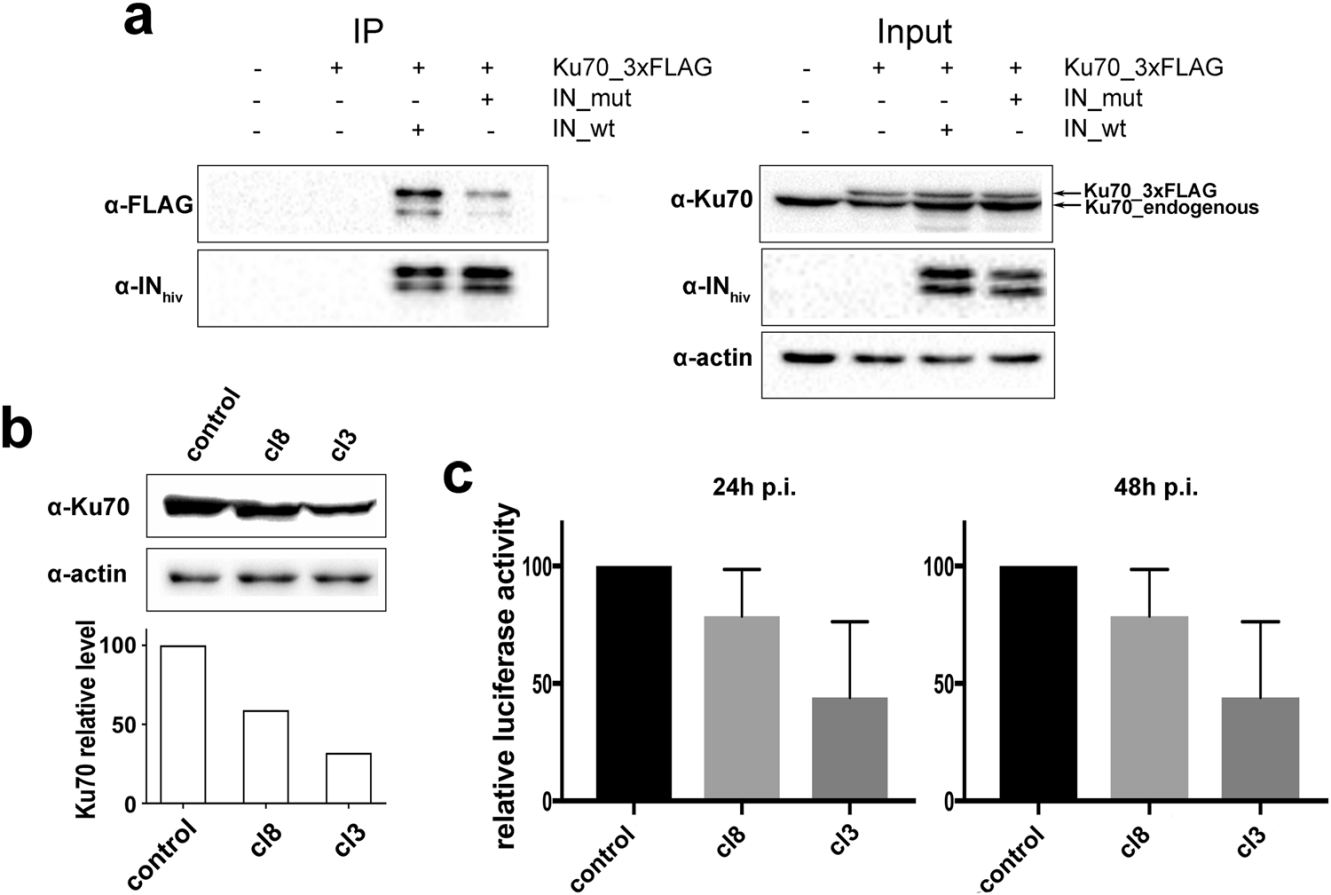
The interaction between Ku70 and IN and the influence of Ku70 on integration in cell culture. (**a**) Immunoprecipitation of an HA-tagged IN_WT or IN_Mut with E212A/L213A substitutions co-transfected in the HEK293T cells together with Ku70_3xFLAG. Eluates were analyzed by Western blot (left panel) with anti-FLAG antibodies to detect Ku70_3FLAG and anti-IN antibodies to detect HIV IN. 10% of the cell lysates were used for input analysis by Western blot (right panel) with anti-Ku70 antibodies for the detection of Ku70 (Ku70_3xFLAG migrates slightly slower than endogenous Ku70) and anti-actin detection to be used as loading control. Cropped images are presented. Uncropped images are shown on Fig. S9. (**b**) Western blot analysis of Ku70 protein levels in HEK 293 T clones expressing either control no-target shRNA (control) or Ku70-targeting shRNA (clone 8 and clone 3). (**c**) Relative luciferase activity in control cells or two clones with reduced Ku70 levels transduced with luciferase expressing replication incompetent HIV-based vector 24 h (left panel) or 48 h (right panel) prior to analysis. Mean values and SDs of three independent experiments are shown.

To reveal the biological significance of our findings, we studied whether substitutions of residues E212 and L213 influence the IN ability to bind Ku70 in cells using conditions described in the work by Zheng *et al*.^29^. For this purpose we used plasmid vectors for eukaryotic expression of wt IN (IN_WT) and IN containing both mutations E212A and L213A, preventing its binding with Ku70 *in vitro* (IN_Mut). Both IN proteins contained a C-terminal HA tag. In addition a plasmid vector for eukaryotic expression of Ku70 with 3xFLAG tag on its C-terminus was used. HEK293T cells were cotransfected either with IN_WT and Ku70 or with IN_Mut and Ku70, and the cell lysates were immunoprecipitated on an anti-HA antibody conjugated agarose (Fig. 8a). The super expressed FLAG-tagged Ku70 was readily co-precipitated with IN_WT whereas its binding with IN_Mut was significantly reduced. We can therefore conclude that amino acids E212 and L213 are crucial for IN binding with Ku70 both *in vitro* and in cell culture.

### Early steps of HIV-1 replication are repressed in cells with reduced Ku70 level

We tested the effect of a reduced intracellular Ku70 concentration on integration. For this purpose we used a single round HIV-based luciferase reporter vector, in which luciferase was placed under the control of the cytomegalovirus (CMV) promoter^44^. This system allowed us to study the effect of Ku70 on early steps of viral replication and particularly integration since transcription from the CMV promoter does not depend on the level of Ku expression^23^. We developed two HEK 293 T based cell lines with a stably knocked down Ku70 expression, one showing a 40–50% reduction in Ku70 protein level (clone 8) while the other with a more pronounced 60–70% reduction (clone 3) (Fig. 8b). For this purpose we used two vectors expressing shRNAs targeting Ku70. A cell line transfected with vector expressing a no-target shRNA was used as a control. These three cell lines were simultaneously transduced and the luciferase expression was assayed either 24 h or 48 h after transduction. Our results show that in cell lines with reduced Ku70 level the luciferase expression, which marks the amount of integrated viral DNA, is weakened and this effect is more pronounced in clone 3 where Ku70 level is lower than in clone 8 (Fig. 8c). This result once again points to a positive role of Ku70 during early stages of HIV replication. However, further research has to be performed to clearly determine which stage of the early steps of replication is affected.

## Discussion

Multiple studies show an importance of the Ku protein for a successful HIV-1 replication^18, 19, 23, 24, 28^. In particular, an interaction between viral IN and Ku70 subunit is found to protect IN from proteasomal degradation, while a reduced amount of Ku70 in the cell weakens integration efficiency^29^. This observation is favored by a report^5^ showing a direct interaction between Ku70 and IN in a yeast two-hybrid assay. Ku is composed of two subunits, Ku70 and Ku80, and a decrease in the cellular level of one subunit leads to the concomitant reduction of the second one. For this reason, an influence of Ku protein on HIV-1 replication is often deduced from data obtained from cells knocked out/down of one subunit only^20, 22, 29, 44, 45^. It has been shown that the knockout of Ku80 does not influence the efficiency of viral DNA integration^23^, an observation that lead to a conclusion that the interaction between IN and Ku70 is irrelevant for a successful integration. However, multiple reports show that Ku subunits may perform independent cellular functions, and a depletion of one subunit does not fully suppress the functioning of the other^46–48^. Interestingly, gel-filtration and cross-linking experiments show that in cells Ku70 in involved in the formation of several macromolecular complexes, with only one of them containing Ku80 as well^48^. Of note, a knock out/down of one subunit may also have an indirect effect on viral integration as Ku participates in a multitude of essential cellular processes^11, 13, 14^. Finally, an interaction between Ku and HIV-1 IN detected in cellular lysates and yeast two-hybrid assay^5, 29^ could be indirect and rather mediated by other proteins. However, a selection of new targets for antiretroviral therapy requires an existence of direct protein-protein binding with a precise data on interaction sites. Then the blocking of these sites could be used to study the effect of these interactions on the viral replication.

Considering this, we studied direct interactions of recombinant Ku70 and IN together with the interactions between their deletion and point mutants using GST or His_6_-pull down assays. All the data obtained are summarized in Table 1. For the first time we demonstrated that Ku70 indeed forms a stable complex with HIV-1 IN and does not bind IN from a different subfamily of Retroviridae, namely PFV IN (Fig. 2). A comparative analysis of these protein structures showed that major differences are located in the helix α6 (a.a. 196–220), therefore it could be the region in the HIV-1 IN responsible for Ku70 binding.

**Table 1:** Wild type and mutant proteins used in the present work and the results of pull-down experiments showing the structural elements important for the IN/Ku70 binding.

IN mutants	Ku70 mutants						
	Ku70	Ku70_1-318	Ku70_1-250	Ku70_251-609	Ku70_251-438	Ku70_1-430	Ku70_439-609
IN_wt	**+**	**+**	**+**	**+**			
IN_1-220	**+**		**+**	**+**			
IN_1-160	**+**		**−**	**+**	**+**	**+**	**−**
IN_51-280	**+**		**+**	**+**			
IN_51-220	**+**		**+**	**+**			
IN_51-160	**+**		**−**	**+**			
IN_1-50	**−**		**−**	**−**			
IN_triple_mut1 (T206A/D207A/T210A)			**+**				
IN_triple_mut2 (Q209A/E212A/L213A)			**−**				
IN_triple_mut3 (K211A/K215A/K219A)			**+**				
IN_Q209A	**+**		**+**	**+**			
IN_E212A	**−**		**−**	**+**			
IN_ L213A	**−**		**−**	**+**			
IN_pfv	**−**						

To confirm the role of the helix α6, we expressed a set of deletion mutants of Ku70 and IN and studied their interactions. According to our data, an isolated N-terminal α/β domain of Ku70 (a.a. 1–250) is sufficient for the binding to IN. The Ku70_1-250 mutant showed a comparable affinity towards both IN and IN_1-220 but failed to bind IN_1-160. This result proved our initial hypothesis that the helix α6 of IN is involved in the binding to Ku70 and particularly interacts with its N-terminal domain. However, Ku70 was still efficient in the binding to IN_1-160 mutant that failed to bind with Ku70_1-250. This data suggests the presence of a second binding site between two proteins. Analysis of interactions between a set of deletion mutants showed that it is the Ku70_251-438 deletion mutant that binds IN_1-160. Also, we showed that both the N- and the C-terminal domains of IN are inessential for complex formation. Two conclusions were made based on this data: 1) the catalytic domain of IN (residues 51–220) is sufficient for IN/Ku70 complex formation and 2) the binding is secured by two sites: the first one located between IN residues 160–220 is responsible for the binding with the Ku70 N-terminal domain while the second is formed by IN residues 50–160 and interacts with the central region of Ku70 (a.a. 251–438).

Our results convincingly confirm the two-site binding mode for IN and Ku70, which was first proposed by Y. Zheng *et al*.^29^, but clarify the regions of both proteins involved in the binding. Moreover, using a set of point mutants we could precisely locate the first binding site within IN. We showed that substitutions T206A, D207A, Q209A, T210A, K211A, K215A and K219A did not affect the protein binding whereas E212A and L213A substitutions have a severe decreasing effect on complex formation between IN and both Ku70_1-250 and Ku70 but show no influence on the interaction with Ku_251-609 containing the second binding site only. Thus we additionally confirmed the two-site mode of IN/Ku70 binding and demonstrated that the residues E212 and L213 are crucial for the protein binding and complex stability. The importance of these residues for the IN/Ku70 interactions was also confirmed by cellular experiments showing that overexpressed IN containing mutations E212A and L213A significantly forfeits its ability to bind Ku70.

The decisive role of the first binding site was reaffirmed using the IN inhibitor 11-OM-E (the conjugate of an 11-mer 2′-O-methyl oligonucleotide with eosin-Y)^42, 43^. Molecular simulations of its interaction with IN region 206–270 a.a. showed that eosin-Y can bind within the C-terminal part of IN that directs the oligonucleotide part of 11-OM-E to shield E212 and L213 residues. The fact that 11-OM-E effectively inhibits the IN/Ku70 interaction additionally proves that the first site plays a key role in the stabilization of IN/Ku70 complex and any changes within this site interfere with complex formation. These data open the perspective to develop small molecule inhibitors of the IN/Ku70 binding using compounds that block residues E212 and L213 in IN. An effective inhibitor of an interaction between Ku70 and IN can not only serve as a perspective therapeutics but can also help to understand the role of this interaction in the HIV-1 replication.

## Materials and Methods

### Oligonucleotides and plasmids

All oligonucleotides (Table S2) were synthesized by the phosphoramidite method. 11-OMe and 11-OMe-E oligonucleotides were synthesized as described earlier^43^. The preparation of plasmid constructs pGEX-6p-1-Ku70, pGEX-6p-1-Ku70_1-250, pGEX-6p-1-Ku70_1-430, pGEX-6p-1-Ku70_251-609, pGEX-6p-1-Ku70_251-609 and pGEX-6p-1-Ku70_439-609 was described in ref. 49. Site directed mutagenesis was performed using Quick Change II Site-Directed Mutagenesis Kit, Agilent Technologies, USA. Plasmid construct pGEX-6p1-Ku70_1-318 was generated from pGEX-6p-1-Ku70 construct by the addition of a STOP-codon by site-directed mutagenesis with primers Ku70-319_STOP/Ku70-319_STOP_anti. Plasmid constructs pET-15b-IN_wt и pET-15b-IN_pfv coding for full-length INs from HIV-1 and PFV, respectively, were kindly provided by Dr. J-F. Mouscadet (ENS de Cachan, France). Constructs pET-15b-IN_1-50, pET-15b-IN_1-220 and pET-15b-IN_1-160 were prepared from pET-15b-IN_wt by the addition of a STOP-codon by site-directed mutagenesis with primer pairs IN_51-STOP/IN_51-STOP_anti, IN_221-STOP/IN_221-STOP_anti, and IN_161-STOP/IN_161-STOP_anti, respectively. Constructs pET-15b-IN_51-280, pET-15b-IN_51-220 and pET-15b-IN_51-160 were produced from pET-15b-IN_wt, pET-15b-IN_1-220 and pET-15b-IN_1-160, respectively, by the addition of NdeI-site before a triplet coding for residue 51 of the corresponding plasmid using primer pair IN_50-NDEI/IN_50-NDEI_anti by site-directed mutagenesis. NdeI (Thermo Scientific, Lithuania) digestion of plasmids carrying an additional NdeI site results in the excision of a fragment coding for IN a.a. 1–50. NdeI predigested and gel purified linearized plasmids were ligated by T4 DNA ligase (Thermo Scientific, Lithuania). Plasmids coding for triple or single IN mutants pET-15b-IN_206/207/210, pET-15b-IN_209/212/213, pET-15b-IN_211/215/219, pET-15b-IN_209, pET-15b-IN_212 and pET-15b-IN_213 were prepared from the construct pET-15b-IN_wt by site-directed mutagenesis with primer pairs IN_206/207/210/IN_206/207/210_anti, IN_209/212/213/IN_209/212/213_anti, IN_211/215/219/IN_211/215/219_anti, IN_209/IN_209_anti, IN_212/IN_212_anti and IN_213/IN_213_anti, respectively. Plasmid vectors pCDNA3_Ku70_3xFLAG and pCDNA3_IN_HA were described earlier^50^. Plasmid pCDNA3_INmut_HA was obtained from pCDNA3_IN_HA by site-directed mutagenesis with primer pairs IN_eu_212/213/IN_eu_212/213_anti.

### Recombinant proteins expression and purification

All HIV-1 and PFV integrases carrying an N-terminal His_6_-tag were expressed and purified as previously described^51^. All Ku70 proteins carrying N-terminal GST-tag and GST alone were purified as previously described^49^. A preformed complex of IN/LEDGFp75 was purified as described in^52^.

### Protein binding assays

To detect interactions between IN and Ku70 or their mutants, the GST-pull-down and His_6_-pull-down assays were performed. IN and Ku70 were incubated in 150 µL of buffer A (20 mM Hepes pH 7.5, 100 mM NaCl, 7.5 mM MgCl_2_, 2 mM 2-merkaptoethanol, 50 µg/ml BSA and 0.1% NP40, in case of His_6_-pull-down 30 mM imidazole was added) at room temperature for 1 hour. Then 20 µL of glutathione-agarose (for GST-pull-down) or Ni-NTA-agarose beads (for His_6_-pull-down) were added to the reaction mixtures followed by 1-hour incubation at room temperature under rotation. Beads were washed twice with washing buffer (buffer A without BSA). The proteins were eluted from the beads with 20 µL of 1X SDS-PAGE loading buffer at 95 °C for 5 minutes and analyzed by SDS-PAGE with subsequent Western blotting. In parallel a non-specific binding of the prey protein was analyzed by the addition of GST-tagged Ku70 samples to Ni-NTA-agarose or His_6_-tagged IN proteins to glutathione-agarose without the presence of respective bait. The pull-down of an individual GST protein was used as a control for a non-specific IN binding to GST.

To investigate the influence of 11-OM-E and 11-OM on the Ku70/IN complex stability, 200 nM IN was incubated with 100 nM Ku70 protein or its mutant Ku70_1-250 as described above and then indicated oligonucleotides were added to the reaction mixture in increasing concentrations (0–10000 nM). Then the level of an IN/Ku70 complex was analyzed by GST-pull-down as described above.

### Western blot analysis

Protein samples were separated by 12% SDS PAGE and analyzed for the presence of GST- or His_6_-tag by WB with rabbit anti-GST (Sigma) and mouse anti-His_6_ antibodies (Sigma), respectively. LEDGF/p75 was detected by mouse anti-LEDGF monoclonal antibody (Santa Cruz Biotechnology). For the detection of IN_HA, an anti-integrate rabbit serum was used (a kind gift from Dr. M. Isaguliants). For the detection of Ku70_3FLAG, an anti-FLAG M2 HRP-conjugated antibody (Sigma) or anti-Ku70 rabbit antibody (Sigma) were used. HRP-conjugated anti-rabbit (Sigma) and anti-mouse antibodies (Sigma) were used as secondary antibodies. Visualization of specific protein bands was performed with Clarity Western ECL substrate (Bio-Rad) on ChemiDoc MP system (Bio-Rad).

### IN catalytic activity tests

The activity of IN_wt and its single point mutants in 3′-processing and strand transfer reactions was analyzed using ^32^P-labeled synthetic DNA substrate as previously described^41^. Briefly, 5 nM ^32^P-labeled duplex U5B/U5A for 3′-processing and 10 nM U5B-2/U5A substrate for strand transfer was incubated with 0, 50 or 100 nM protein in 20 µL of reaction buffer (20 mM Hepes pH 7.2, 1 mM DTT and 7.5 mM MgCl_2_) for 2 h at 37 °C. The reaction products were separated by electrophoresis in a 20% polyacrylamide/7 M urea gel. Gel images were recorded on Typhoon FLA9500 Phosphorimager (GE Healthcare, USA).

### Docking and molecular dynamics

Molecular docking was performed with Autodock Vina^53^. Generation of input PDBQT files and processing of outputs were done with AutoDock Tools^54^. Receptor protein structure was generated from PDB ID 1EX4. Chain A was selected and truncated to residues 206–270. Protonation states were assigned matching those at pH 7.0. Structures of ligands (eosin-Y and eosin-Y+linker) were created from initial structures with assigned Gasteiger charges prepared in Avogadro platform^55^. Docking cell included whole fragment with padding of 5 Å from edge atoms. Due to large number of rotational bonds in case of eosin-Y+linker structure “exhaustiveness” parameter was set to 1024 for all systems and 20 independent runs were performed. During docking receptor was kept rigid while ligand was flexible.

Top hits from docking were used as starting points for modeling. Initial structure of 11-OM-E was generated with X3DNA^56^ as an A-form helix and fused with eosin-Y+linker with PyMol^57^. To level drawbacks of rough fusing procedure all possible rotamers for dihedral angle C3′-O3′-P-O (around linkage between last nucleotide and linker) were generated with a step of 20 degrees.

All molecular dynamics simulations were performed with GROMACS 5.1.2^58^ package with amber 99sb-ildn^59^ force field for protein and parmbsc0(chi-OL3)^60^ correction set for RNA and partial charges for 2′-OMe-RNA^61^. Topology for eosin-Y+linker structure was generated with ACPYPE^62^. Each of 36 systems was put in the center of triclinic box with the padding of 1.5 nm from edge atoms. Energy minimization run utilized Steepest descent algorithm with convergence threshold of 100 kJ mol^−1^ nm^−1^ and maximum number of steps up to 1*10^6^ steps. Cut-off scheme was set to Verlet and cut-off parameters for interactions were set to 2.0 nm. VdW scheme was set to LJ-PME^63^.

Simulated-annealing-like procedure included 100 ns molecular dynamics simulation with the step size of 1 fs in vacuum and electrostatic interactions as main acting forces. Initial velocities were independently generated for every run. Protein and oligonucleotide were divided into two temperature coupling groups with reference temperatures of 10 K and 400 K respectively with V-rescale as thermostat. Cut-off scheme was set to Cut-off with cut-off values same as in energy minimization. Simulation procedure was repeated 5 times for every rotamer, which successfully passed the EM procedure. Minimal distances between atoms in oligonucleotide and amino acid were measured and plotted with a set of Python^64^ tools: ProDy^65^, NumPy^66^, Matplotlib^67^. Visualization of biomolecular structures was done in PyMol package.

### Mass-spectrometry

The protein samples were prepared for MS identification and analyzed as described^68^. Mass-spectra were obtained on UltrafleXtreme Bruker Daltonics MALDI-TOF mass-spectrometer, analyzed by FlexAnalysis 3.3 (Bruker Daltonics, Germany), protein identification was performed using Mascot (www.matrixscience.com) (Table S1).

### Cell culture and viral assembly

HEK293T cells were cultured in DMEM medium supplemented with 10% FBS and penicillin/streptomycin solution (all obtained from Invitrogen). Viral vectors were assembled as described in ref. 44. pCMVΔ8.2R viral packaging plasmid and pCMV VSVG plasmid were obtained from Addgene. pUCHR_inLuc genomic plasmid was a kind gift of Dr. D. Mazurov. Viral stocks were concentrated by centrifugation at 30000 g and resuspended in PBS. p24 was assayed using the HIV-1 p24-antigen IFA kit (Vector Best).

### Co-immunoprecipitation

2 × 10^6^ 293 T cells were transfected with 9 μg of empty pCDNA3.1 vector or cotransfected with 3 μg of pCDNA3_Ku70_3xFLAG and 6 μg of pCDNA3_IN_HA/pCDNA3_INmut_HA/pCDNA3.1 vectors using the TurboFect transfection reagent (Thermo Fisher Scientific). 48 h after transfection cells were lysed for 30 min on ice in RPMI medium (Invitrogen) supplemented with Protease inhibitor cocktail (Thermo Fisher Scientific), 10 µM MG132 (Sigma) and 0.25% NP-40 (Helicon). Lysates were cleared by centrifugation for 10 min at 14000 rpm and protein concentration was measured on the NanoDrop 2000 spectrophotometer (Thermo Fisher Scientific). 0.1 mg of cell lysates were saved for input analysis. 1 mg of total protein was mixed with HA-antibody conjugated agarose (Sigma) and incubated for 5 h at 4 °C. The beads were washed 4 times with lysis buffer and bound proteins were eluted with SDS loading buffer for 5 min at 95 °C. Elution fractions and inputs were then analyzed by Western blot (see above).

### Stable knock down of Ku70 and viral vector transduction assay

For the stable knock down of Ku70 two pSuper.retro.puro (Oligoengine) vectors expressing shRNAs targeting two sites in Ku70 were used. Short duplexes coding for shRNA352 (Sen_shKu70_352/Anti_shKu70_352), shRNA1025 (Sen_shKu70_1025/Anti_shKu70_1025) or shRNACntrl (Sen_shCNTRL/Anti_shCNTRL) were cloned between BglII and HindIII sites. 4 × 10^5^ HEK293T cells were co-transfected with 6 µg of each vector targeting Ku70 or with 6 μg of a vector expressing control shRNA, and the transfected cells were selected with 1 µg/mL of puromycin (Cell Signaling Technology). Individual clones were isolated and Ku70 protein expression was tested by Western blot. Two clones with reduced Ku70 protein expression were selected for further use: clone 8 with a 50% and clone 3 with a 75% reduction in Ku70 expression.

2 × 10^5^ cells of clone 8, clone 3 or control clone were transduced with 1 MOI of HIV-1 viral vector. 24 h or 48 h post infection cells were harvested, cell number was counted and luciferase activity in cell lysates was assayed using the Luciferase assay system kit (Promega).

## Electronic supplementary material

Supplementary Information
