# Supplementary material for: Characterization of HIV-1 integrase interaction with human Ku70 protein and initial implications for drug targeting
Source: Sci Rep. 2017 Jul 17;7:5649. doi: 10.1038/s41598-017-05659-5 (PMC5514147; doi:10.1038/s41598-017-05659-5)
Supplement: Supplementary file 1 — Supplementary Information [file 41598_2017_5659_MOESM1_ESM.pdf]

Supplementary material

Characterization of HIV-1 integrase interaction with human Ku70 protein and initial implications for drug targeting

Andrey N. Anisenko[1], Ekaterina S. Knyazhanskaya[*2], Artur O. Zalevsky[1], Julia Yu. Agapkina[2], Aleksander I. Sizov[2], Timofey S. Zatsepin[2,3], Marina B. Gottikh[4]*

[1]Faculty of Bioengineering and Bioinformatics, Lomonosov Moscow State University, Moscow;
[2] Chemistry Department, Lomonosov Moscow State University, Moscow;
[3]Skolkovo Institute of Science and Technology, Skolkovo, Russia;
[4]Belozersky Institute of Physico-Chemical Biology, Lomonosov Moscow State University, Moscow

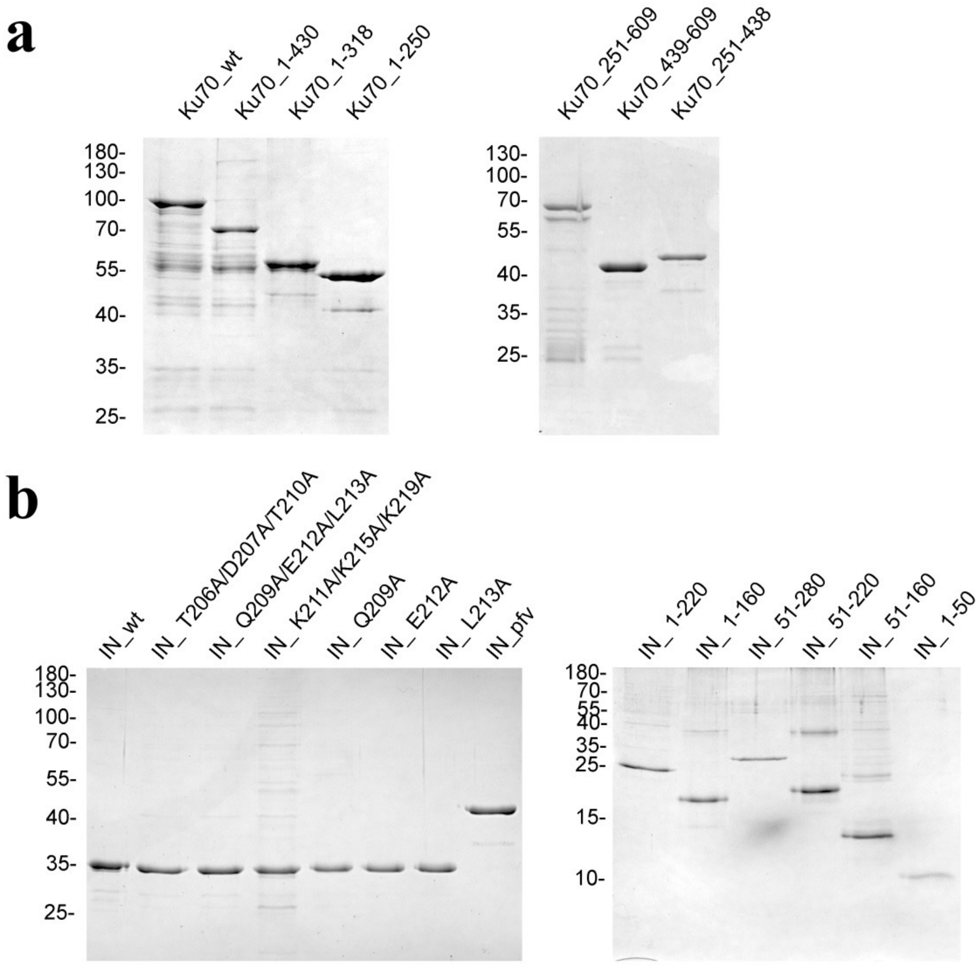

Fig. S1. SDS PAGE analysis of protein preparations used in the work with subsequent Coomassie blue staining. **a**. Full-length Ku70 and its deletion mutants. **b**. PFV IN, HIV-1 IN and a set of HIV-1 IN deletion and point mutants

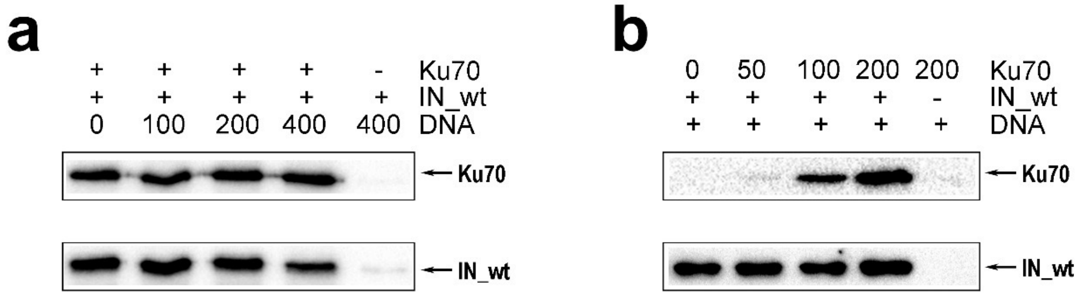

Fig. S2. Effect of the 40-mer DNA mimicking the end of the viral U5 DNA on the IN/Ku70 complex formation. **a.** Binding of IN (200 nM) to Ku70 (100 nM) in the presence of increasing concentrations of DNA analyzed by GST-pull-down. **b.** Formation of the triple IN/Ku70/DNA complex analyzed by biotin-pull-down. Ku70 concentrations (nM) are marked above; IN was taken at 100 nM concentration and biotin-labeled 40-mer DNA was taken at 50 nM.

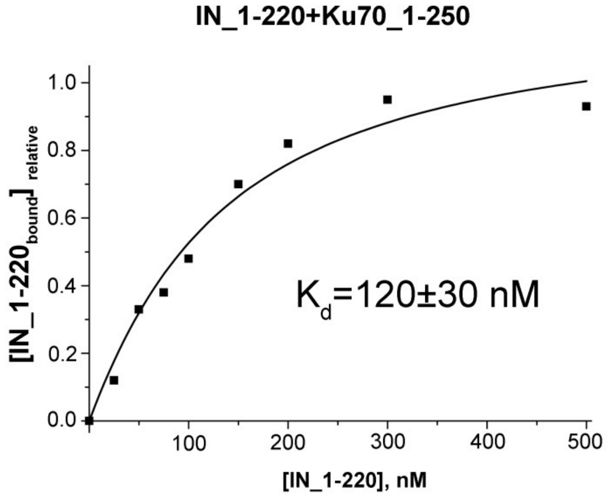

Fig. S3. Titration curve for Ku70_1-250 treated with increasing concentrations of IN_1-220 deletion mutant.

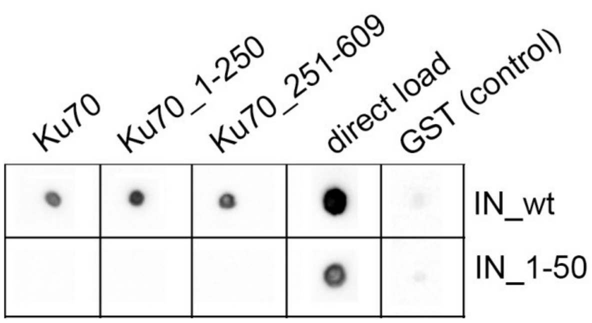

Fig. S4. GST pull-down assay with subsequent dot-blot analysis of the binding between full-length IN (IN_wt) or IN_1-50 (N-terminal domain) and Ku70, Ku70_1-250 or Ku70_251-609.

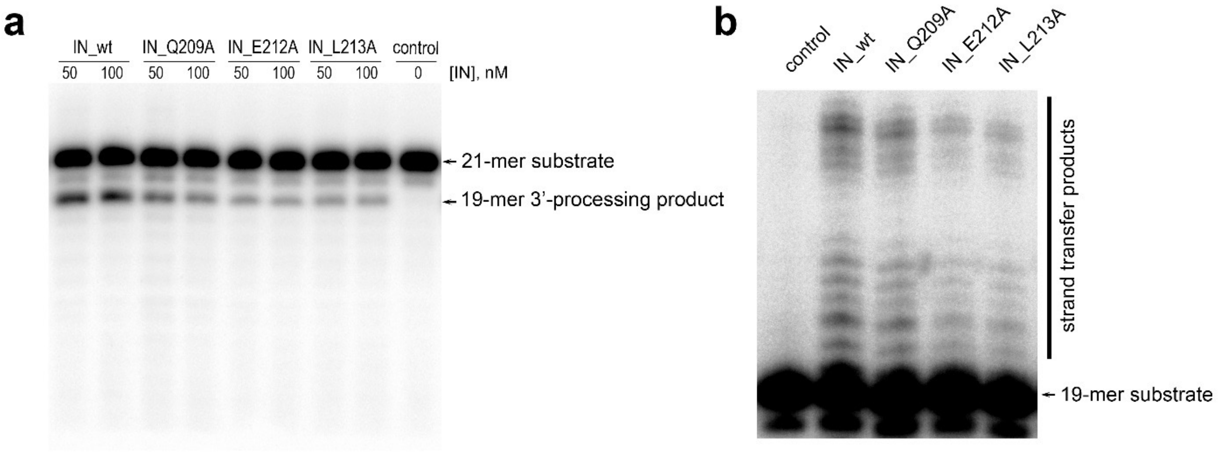

Fig. S5. The catalytic activity of HIV-1 IN and its mutants IN_Q209A, IN_E212A and IN_L213A in 3'-processing (**a**) and strand transfer reactions (**b**). The 3'-processing reaction was performed at 37ºC for 2 h using different IN concentrations (50 and 100 nM) and 5 nM U5B/U5A substrate; for the strand transfer reaction 100 nM IN and 10 nM U5B-2/U5A substrate were used.

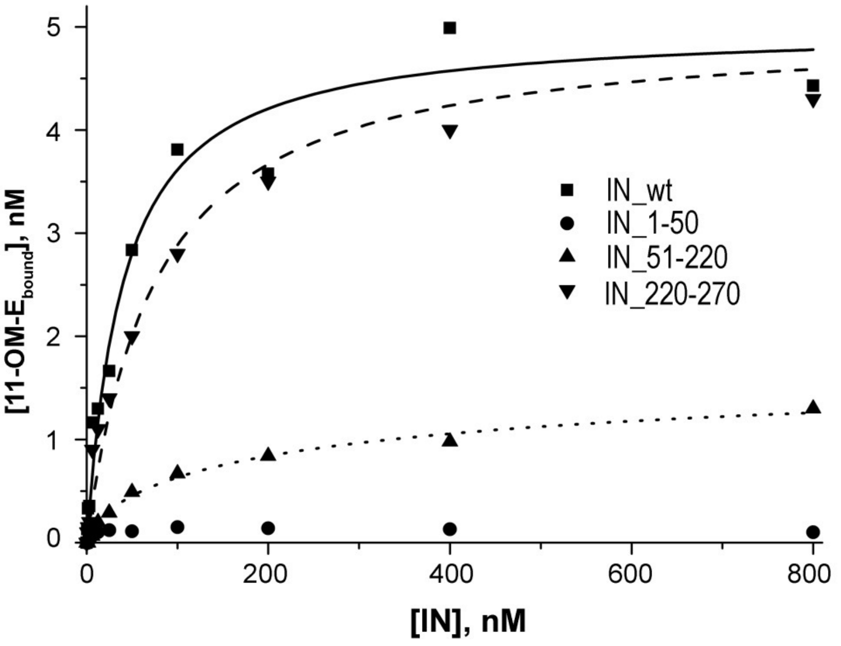

Fig. S6. Binding of the oligonucleotide inhibitor 11-OM-E with IN and its deletion mutants (IN_1-50, IN_51-220, IN_220-270) analyzed by a nitrocellulose binding assay.

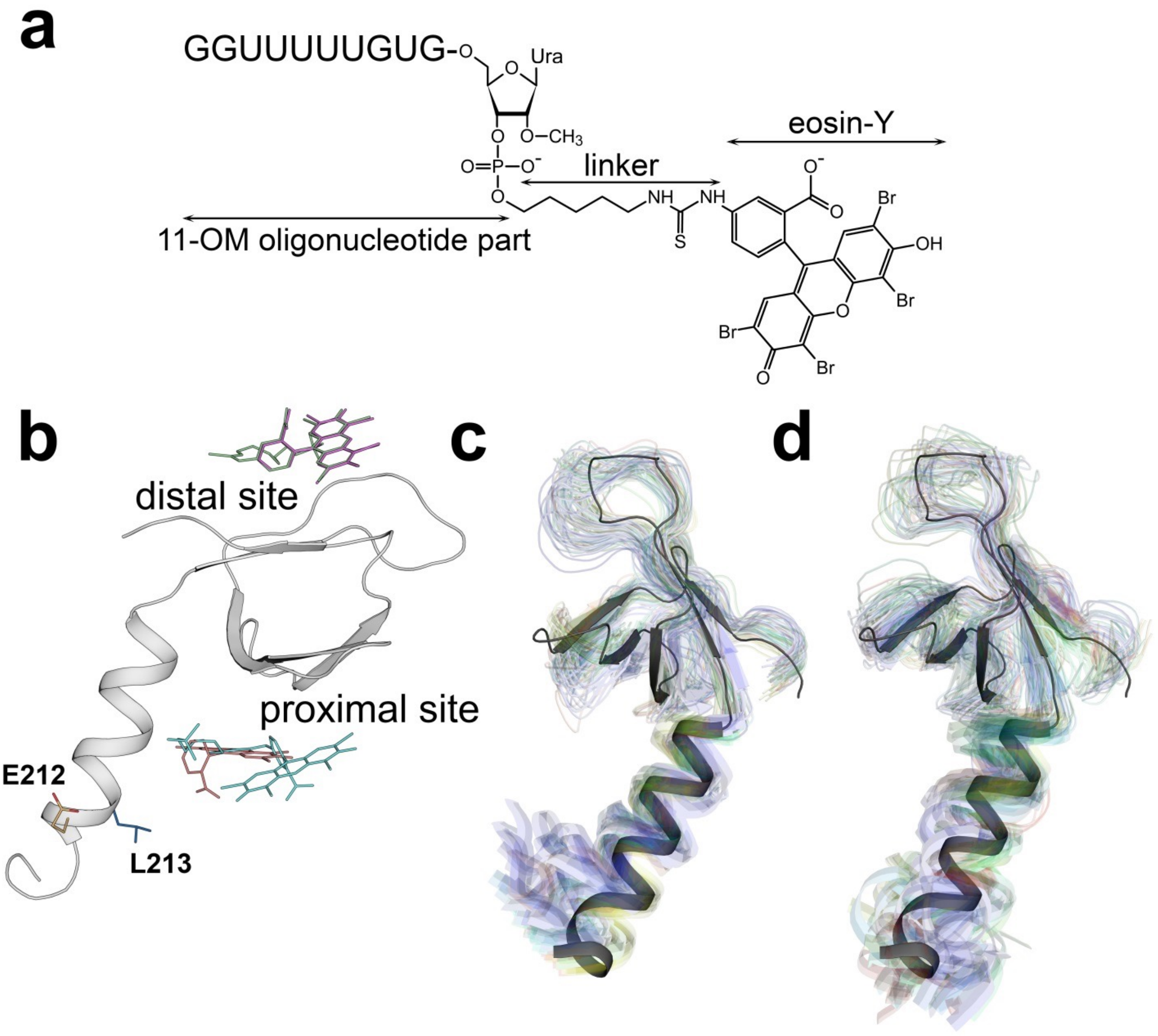

Fig. S7. Structure of the oligonucleotide inhibitor 11-OM-E and its interactions with HIV-1 IN. **a.** The chemical structure of the inhibitor. **b**. Docking sites of eosinY and eosinY+linker in the isolated domain of IN. Distal site is placed at the top and proximal is at the bottom. E212 and L213 are colored in yellow and blue, respectively. **c-d** Molecular dynamics showing conformational changes in the IN region 206-270 a.a. when bound to oligonucleotide-eosine conjugate (hidden). The initial structure of IN bound to 11-OM-E is shown in solid grey. Possible structural changes are shown in transparent blue for eosin bound in distal site (**c**) and in proximal site (**d**).

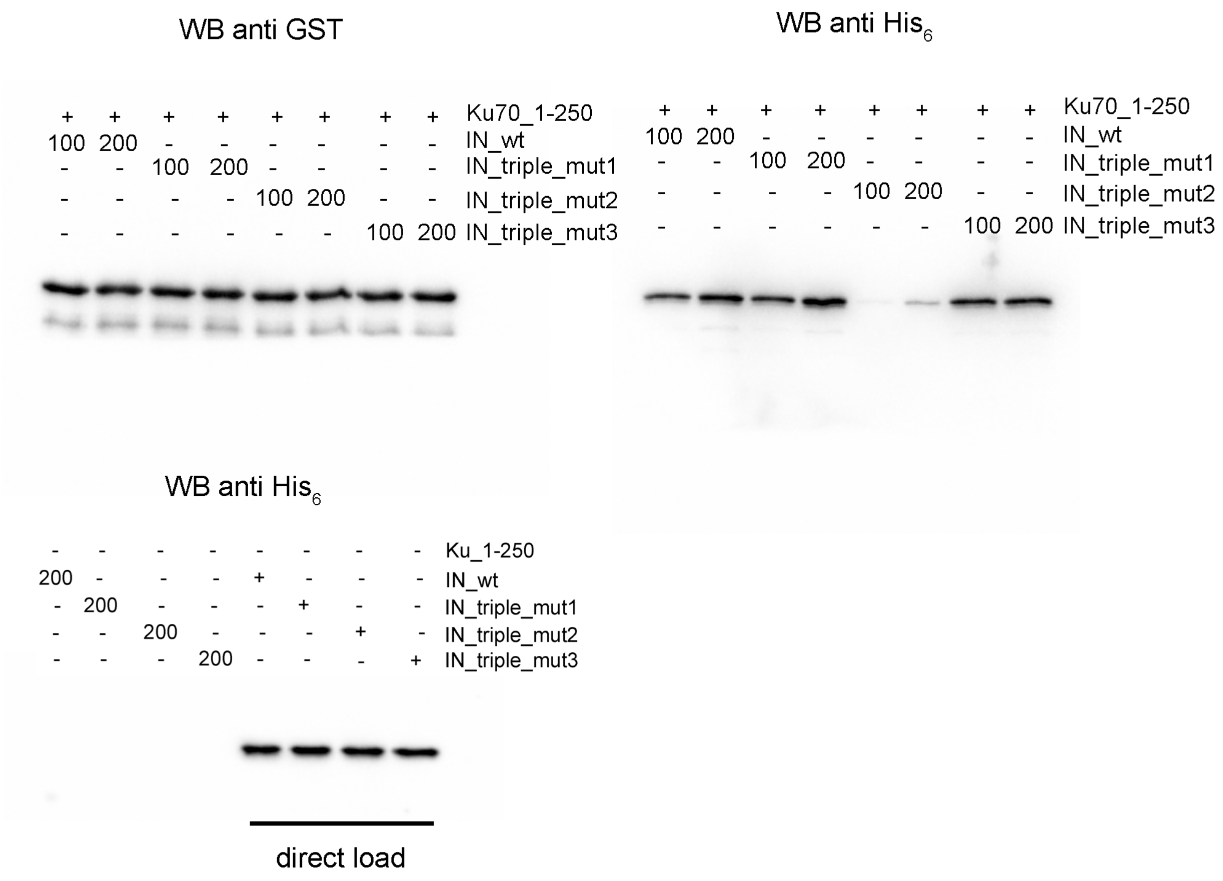

Fig. S8. The influence of mutations in the 200-220 a.a. region of IN on its interaction with Ku70. Uncropped Wester blots of images demonstrated on Fig. 6b in the main text.

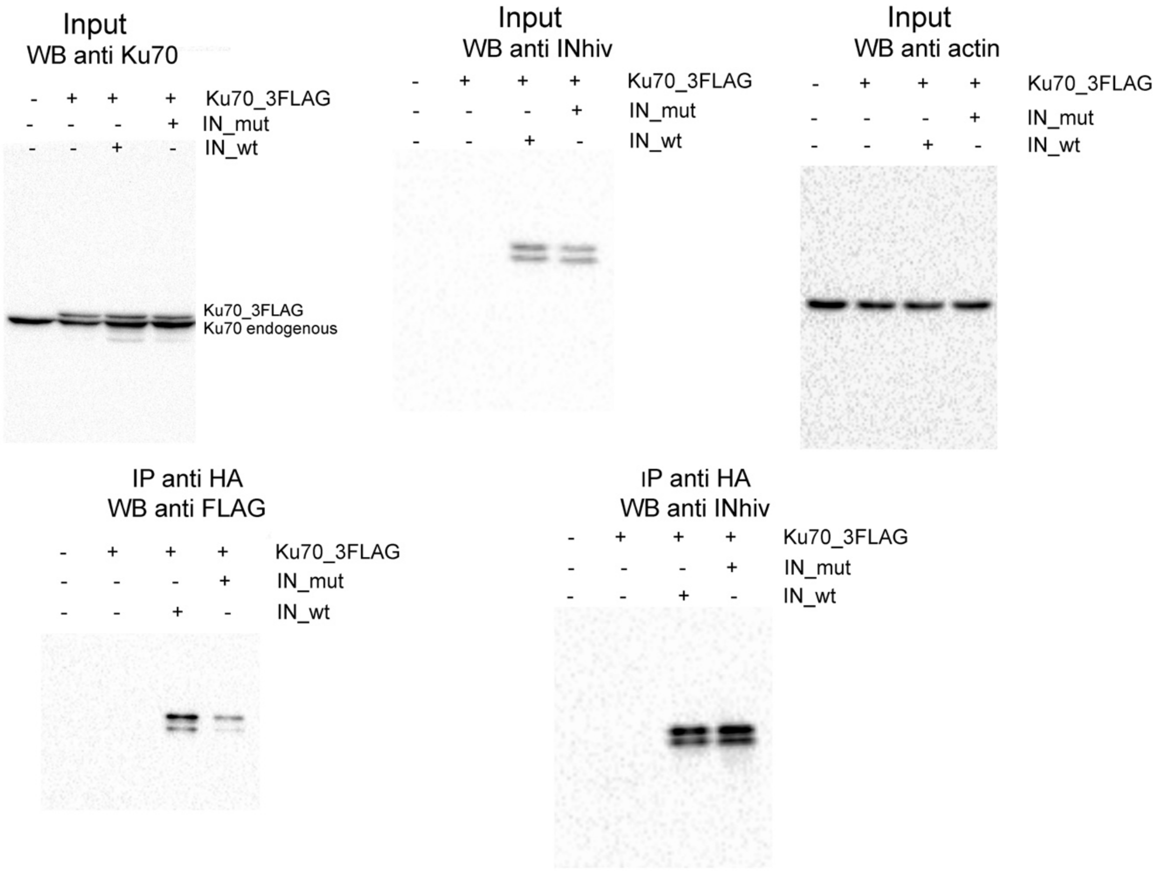

Fig.S9. The interaction between Ku70 and IN in cell culture. Uncropped Western blots images of inputs (lysates of transfected HEK293T cells) and eluates of immunoprecipitation on anti-HA conjugated agarose (for details see main text, Fig. 8a).

Table S1. Lists of peptides observed in mass-spectrometry experiment for trypsin digested GST-Ku70_deg. Amino acids related to GST-tag are marked in red, amino acids related to Ku70 are marked in green.

Start - End, a.a.	Mr(expt)	Mr(calc)	Delta	Sequence	Variable modification
1 - 9	1093.5689	1093.5630	0.0059	MSPILGYWK	
1 - 9	1109.5625	1109.5579	0.0046	MSPILGYWK	Oxidation (M)
2 - 9	962.5220	962.5225	-0.0005	SPILGYWK	
12 - 18	769.4559	769.4446	0.0113	K.GLVQPTR	
19 - 27	1148.6376	1148.6328	0.0048	LLLEYLEEK	
19 - 42	3154.5276	3154.5247	0.0029	LLLEYLEEKYEEHLYERDEGDKWR	
28 - 35	1137.5198	1137.5090	0.0107	YEEHLYER	
28 - 42	2023.9309	2023.9024	0.0286	YEEHLYERDEGDKWR	
36 - 42	904.4082	904.4039	0.0043	DEGDKWR	
45 - 64	2356.2311	2356.1991	0.0320	KFELGLEFPNLPYYIDGDVK	
46 - 64	2228.1283	2228.1041	0.0242	FELGLEFPNLPYYIDGDVK	
65 - 73	1031.5933	1031.5797	0.0136	LTQSMAIIR	
65 - 73	1047.5742	1047.5746	-0.0005	LTQSMAIIR	Oxidation (M)
65 - 78	1621.7613	1621.8861	-0.1248	LTQSMAIIRYIADK	
74 - 87	1616.8074	1616.7803	0.0271	YIADKHNMLGGCPK	Propionamide (C)
74 - 89	1901.8943	1901.9240	-0.0297	YIADKHNMLGGCPKER	Propionamide (C)
74 - 89	1917.8934	1917.9189	-0.0255	YIADKHNMLGGCPKER	Oxidation (M), Propionamide (C)
88 - 103	1800.9717	1800.9403	0.0313	ERAEISMLEGAVLDIR	
90 - 103	1515.8321	1515.7966	0.0354	AEISMLEGAVLDIR	
90 - 103	1531.8110	1531.7916	0.0194	AEISMLEGAVLDIR	Oxidation (M)
109 - 113	580.3576	580.3220	0.0355	IAYSK	
109 - 119	1313.6945	1313.6867	0.0079	IAYSKDFETLK	
114 - 119	751.3863	751.3752	0.0111	DFETLK	
114 - 125	1440.7730	1440.7500	0.0230	DFETLKVDFLSK	
114 - 131	2152.0686	2152.1490	-0.0803	DFETLKVDFLSKLPEMLK	
120 - 125	707.3847	707.3854	-0.0007	VDFLSK	
126 - 131	729.4226	729.4095	0.0131	LPEMLK	
132 - 136	696.3100	696.2901	0.0199	MFEDR	
132 - 140	1177.5477	1177.5372	0.0105	MFEDRLCHK	

176 - 180	679.3950	679.3727	0.0222	LVCFK	Propionamide (C)
181 - 191	1309.7901	1309.7717	0.0183	KRIEAIPQIDK	
182 - 191	1181.6990	1181.6768	0.0222	RIEAIPQIDK	
183 - 191	1025.5769	1025.5757	0.0012	IEAIPQIDK	
198 - 218	2325.1805	2325.1331	0.0474	YIAWPLQGWQATFGGGDHPPK	
219 - 239	2361.1657	2361.1165	0.0492	SDLEVLFQGPLGSSGWESYYK	
240 - 261	2500.0070	2499.9884	0.0186	TEGDEEAEEEQEENLEASGDYK	
266 - 276	1206.6646	1206.6496	0.0150	DSLIFLVDASK	
305 - 322	2025.1231	2025.0782	0.0449	IISSDRDLLAVVFYGTEK	
305 - 324	2268.2127	2268.2002	0.0125	IISSDRDLLAVVFYGTEKDK	
305 - 330	2957.5652	2957.5498	0.0154	IISSDRDLLAVVFYGTEKDKNSVNFK	
311 - 322	1353.7327	1353.7180	0.0147	DLLAVVFYGTEK	
311 - 324	1596.8622	1596.8399	0.0223	DLLAVVFYGTEKDK	
323 - 330	950.4755	950.4821	-0.0067	DKNSVNFK	
331 - 344	1572.8452	1572.8147	0.0305	NIYVLQELDNPGAK	
331 - 345	1728.9498	1728.9158	0.0340	NIYVLQELDNPGAKR	
345 - 353	1160.6658	1160.6553	0.0105	RILELDQFK	
346 - 353	1004.5542	1004.5542	-0.0000	ILELDQFK	
346 - 359	1630.8917	1630.8679	0.0239	ILELDQFKGQQGQK	
354 - 359	644.3642	644.3242	0.0401	GQQGQK	
391 - 395	657.3656	657.3380	0.0276	MSHKR	
395 - 412	2058.0014	2057.9589	0.0425	RIMLFTNEDNPHGNDSAK	
420 - 424	530.3698	530.2813	0.0885	AGDLR	
425 - 436	1401.7733	1401.7326	0.0407	DTGIFLDLMHLK	
425 - 436	1417.7568	1417.7275	0.0292	DTGIFLDLMHLK	Oxidation (M)
437 - 448	1398.7606	1398.7296	0.0310	KPGGFDISLFYR	
449 - 460	1387.6987	1387.6831	0.0156	DIISIAEDEDLR	
449 - 468	2331.1773	2331.1230	0.0543	DIISIAEDEDLRVHFEESSK	
449 - 474	3070.5462	3070.5458	0.0004	DIISIAEDEDLRVHFEESSKLEDLLR	
461 - 474	1700.9150	1700.8733	0.0417	VHFEESSKLEDLLR	
469 - 474	757.4481	757.4334	0.0147	LEDLLR	
478 - 482	603.3882	603.3340	0.0543	AKETR	

510 - 517	862.5651	862.5640	0.0011	ALKPPPIK	
532 - 547	1650.8775	1650.8465	0.0310	TFNTSTGGLLLPSDTK	
532 - 548	1807.0056	1806.9476	0.0580	TFNTSTGGLLLPSDTKR	

Table S2. Oligonucleotides used for preparation of prokaryotic and eukaryiotic protein expression vectors, shRNA expressing vectors and for measurements of integrase 3'-processing activity.

Name	Oligonucleotide sequence (5'-3')
Ku70-319_STOP	GACGACTCCCATAGATCTGCTACCTCTTGGTATCGCTAGG
Ku70-319_STOP_anti	CCTAGCGATACCAAGAGGTAGCAGATCTATGGGAGTCGTC
IN_221-STOP	TGTCCCTGTAATAAACCCGAAAATTTTGCTATTTTGTAATTTGTTTTTGTAATTCTTTAGTTTGTATGTCT
IN_221-STOP_anti	AGACATACAAACTAAAGAATTACAAAAACAAATTACAAAATAGCAAAATTTTCGGGTTTATTACAGGGACA
IN_161-STOP	CCTGATCTCTTACCTGTCCTATAATTTACTTTAATTCTTTATTCATAGATTCTAT
IN_161-STOP_anti	ATAGAATCTATGAATAAAGAATTAAAGTAAATTATAGGACAGGTAAGAGATCAGG
IN_51-STOP	GGCTACAGTCTACTTGTCCCTACATGGCTTCCCCTTTTAGC
IN_51-STOP_anti	GCTAAAAGGGGAAGCCATGTAGGGACAAGTAGACTGTAGCC
IN_50-NDEI	AGCTAAAAGGGGAAGCCATATGCATGGACAAGTAGA
IN_50-NDEI_anti	TCTACTTGTCCATGCATATGGCTTCCCCTTTTAGCT
IN_211/215/219	GCTGTCCCTGTAATAAACCCGAAAATTTTGAATTGCTGTAATTTGTGCTTGTAATTCTGCAGTTTGTATGTCTGTTGCTATTATGTCTACTATT
IN_211/215/219_anti	AATAGTAGACATAATAGCAACAGACATACAAACTGCAGAATTACAAGCACAAATTACAGCAATTCAAAATTTTCGGGTTTATTACAGGGACAGC
IN_209/212/213	GAAAATTTTGAATTTTTGTAATTTGTTTTTGTGCTGCTTTAGTTGCTATGTCTGTTGCTATTATGTCTACTATTCTTTCCCCTGCACT

IN_209/212/213_anti	AGTGCAGGGGAAAGAATAGTAGACATAATAGCAACAGACATAGCAACTAAAGCAGCACAAAAACAAATTACAAAAATTCAAAATTTTC
IN_206/207/210	TTTTGTAATTTGTTTTTGTAATTCTTTAGCTTGTATGGCTGCTGCTATTATGTCTACTATTCTTTCCCCTG
IN_206/207/210_anti	CAGGGGAAAGAATAGTAGACATAATAGCAGCAGCCATACAAGCTAAAGAATTACAAAAACAAATTACAAAA
IN_209	TTTTTGTAATTTGTTTTTGTAATTCTTTAGTTGCTATGTCTGTTGCTATTATGTCTACTATTCTTTC
IN_209_anti	GAAAGAATAGTAGACATAATAGCAACAGACATAGCAACTAAAGAATTACAAAAACAAATTACAAAAA
IN_212	GACATAATAGCAACAGACATACAAACTAAAGCATTACAAAAACAAATTACAAAAATTCAAAA
IN_212_anti	ATTTTGAATTTTTGTAATTTGTTTTTGTAATGCTTTAGTTTGTATGTCTGTTGCTATTATGT
IN_213	GAAAATTTTGAATTTTTGTAATTTGTTTTTGTGCTTCTTTAGTTTGTATGTCTGTTGCTATTATGTCTACTAT
IN_213_anti	ATAGTAGACATAATAGCAACAGACATACAAACTAAAGAAGCACAAAAACAAATTACAAAAATTCAAAATTTTC
U5B	GTGTGGAAAATCTCTAGCAGT
U5B-2	GTGTGGAAAATCTCTAGCA
U5A	ACTGCTAGAGATTTTCCACAC
Sen_shKu70_352	GATCCCCGTGCAAAACGAATTCTAGATTCAAGAGATCTAGAATTCGTTTTGCACTTTTTA
Anti_shKu70_352	AGCTTAAAAAGTGCAAAACGAATTCTAGATCTCTTGAATCTAGAATTCGTTTTGCACGGG
Sen_shKu70_1025	GATCCCCGCTAAAACGGTTTGATGATTTCAAGAGAATCATCAAACCGTTTTAGCTTTTTA
Anti_shKu70_1025	AGCTTAAAAAGCTAAAACGGTTTGATGATTCTCTTGAAATCATCAAACCGTTTTAGCGGG
Sen_shCNTRL	GATCCCCGTCACGTCTAACTAACTAAGAACCTTGTTCAAGAGACAAGGTTCTTAGTTAGTTAGACGTGACTTTTTA

Anti_shCNTRL	AGCTTAAAAAGTCACGTCTAACTAACTAAGAACCTTGTCTCTTGAACAAGGTTCTTAGTTAGTTAGACGTGACGGG
IN_eu_212/213	GAAAATTTTGAATTTTTGTAATTTGTTTTTGTGCTGCTTTAGTTTGTATGTCTGTTGCTATTATGT
IN_eu_212/213_anti	ACATAATAGCAACAGACATACAAACTAAAGCAGCACAAAAACAAATTACAAAAATTCAAAATTTTC
